# Longitudinal imaging highlights preferential basal ganglia circuit atrophy in Huntington’s disease

**DOI:** 10.1093/braincomms/fcad214

**Published:** 2023-08-18

**Authors:** Chin-Fu Liu, Laurent Younes, Xiao J Tong, Jared T Hinkle, Maggie Wang, Sanika Phatak, Xin Xu, Xuan Bu, Vivian Looi, Jee Bang, Sarah J Tabrizi, Rachael I Scahill, Jane S Paulsen, Nellie Georgiou-Karistianis, Andreia V Faria, Michael I Miller, J Tilak Ratnanather, Christopher A Ross

**Affiliations:** Center for Imaging Science, Johns Hopkins University, Baltimore, MD 21218, USA; Institute for Computational Medicine, Johns Hopkins University, Baltimore, MD 21218, USA; Department of Biomedical Engineering, Johns Hopkins University, Baltimore, MD 21218, USA; Center for Imaging Science, Johns Hopkins University, Baltimore, MD 21218, USA; Institute for Computational Medicine, Johns Hopkins University, Baltimore, MD 21218, USA; Department of Applied Mathematics and Statistics, Johns Hopkins University, Baltimore, MD 21218, USA; Division of Neurobiology, Department of Psychiatry, Johns Hopkins University School of Medicine, Baltimore MD 21287, USA; Department of Neuroscience, Johns Hopkins University School of Medicine, Baltimore, MD 21218, USA; Medical Scientist Training Program, Johns Hopkins University School of Medicine, Baltimore, MD 21287, USA; Center for Imaging Science, Johns Hopkins University, Baltimore, MD 21218, USA; Department of Biomedical Engineering, Johns Hopkins University, Baltimore, MD 21218, USA; Center for Imaging Science, Johns Hopkins University, Baltimore, MD 21218, USA; Department of Biomedical Engineering, Johns Hopkins University, Baltimore, MD 21218, USA; Division of Magnetic Resonance, Department of Radiology, Johns Hopkins University School of Medicine, Baltimore, MD 21287, USA; Center for Imaging Science, Johns Hopkins University, Baltimore, MD 21218, USA; Institute for Computational Medicine, Johns Hopkins University, Baltimore, MD 21218, USA; Huaxi MR Research Center, Department of Radiology, West China Hospital of Sichuan University, Chengdu, Sichuan 610041, China; Center for Imaging Science, Johns Hopkins University, Baltimore, MD 21218, USA; Institute for Computational Medicine, Johns Hopkins University, Baltimore, MD 21218, USA; Department of Biomedical Engineering, Johns Hopkins University, Baltimore, MD 21218, USA; Division of Neurobiology, Department of Psychiatry, Department of Neurology, Johns Hopkins University School of Medicine, Baltimore, MD 21287, USA; HD Research Centre, University College London Queen Square Institute of Neurology, UCL, London, UK; HD Research Centre, University College London Queen Square Institute of Neurology, UCL, London, UK; Department of Neurology, University of Wisconsin, Madison, WI 53705, USA; School of Psychological Sciences and The Turner Institute for Brain and Mental Health, Monash University, Melbourne, Victoria 3800, Australia; Division of Magnetic Resonance, Department of Radiology, Johns Hopkins University School of Medicine, Baltimore, MD 21287, USA; Center for Imaging Science, Johns Hopkins University, Baltimore, MD 21218, USA; Institute for Computational Medicine, Johns Hopkins University, Baltimore, MD 21218, USA; Department of Biomedical Engineering, Johns Hopkins University, Baltimore, MD 21218, USA; Center for Imaging Science, Johns Hopkins University, Baltimore, MD 21218, USA; Institute for Computational Medicine, Johns Hopkins University, Baltimore, MD 21218, USA; Department of Biomedical Engineering, Johns Hopkins University, Baltimore, MD 21218, USA; Division of Neurobiology, Department of Psychiatry, Johns Hopkins University School of Medicine, Baltimore MD 21287, USA; Department of Neuroscience, Johns Hopkins University School of Medicine, Baltimore, MD 21218, USA; Division of Neurobiology, Department of Psychiatry, Department of Neurology, Johns Hopkins University School of Medicine, Baltimore, MD 21287, USA; Department of Pharmacology, Johns Hopkins University School of Medicine, Baltimore, MD 21287, USA

**Keywords:** caudate, putamen, PREDICT-HD, TRACK-HD, IMAGE-HD

## Abstract

Huntington’s disease is caused by a CAG repeat expansion in the Huntingtin gene (*HTT*), coding for polyglutamine in the Huntingtin protein, with longer CAG repeats causing earlier age of onset. The variable ‘Age’ × (‘CAG’—L), where ‘Age’ is the current age of the individual, ‘CAG’ is the repeat length and L is a constant (reflecting an approximation of the threshold), termed the ‘CAG Age Product’ (CAP) enables the consideration of many individuals with different CAG repeat expansions at the same time for analysis of any variable and graphing using the CAG Age Product score as the X axis. Structural MRI studies have showed that progressive striatal atrophy begins many years prior to the onset of diagnosable motor Huntington’s disease, confirmed by longitudinal multicentre studies on three continents, including PREDICT-HD, TRACK-HD and IMAGE-HD. However, previous studies have not clarified the relationship between striatal atrophy, atrophy of other basal ganglia structures, and atrophy of other brain regions. The present study has analysed all three longitudinal datasets together using a single image segmentation algorithm and combining data from a large number of subjects across a range of CAG Age Product score. In addition, we have used a strategy of normalizing regional atrophy to atrophy of the whole brain, in order to determine which regions may undergo preferential degeneration. This made possible the detailed characterization of regional brain atrophy in relation to CAG Age Product score. There is dramatic selective atrophy of regions involved in the basal ganglia circuit—caudate, putamen, nucleus accumbens, globus pallidus and substantia nigra. Most other regions of the brain appear to have slower but steady degeneration. These results support (but certainly do not prove) the hypothesis of circuit-based spread of pathology in Huntington’s disease, possibly due to spread of mutant Htt protein, though other connection-based mechanisms are possible. Therapeutic targets related to prion-like spread of pathology or other mechanisms may be suggested. In addition, they have implications for current neurosurgical therapeutic approaches, since delivery of therapeutic agents solely to the caudate and putamen may miss other structures affected early, such as nucleus accumbens and output nuclei of the striatum, the substantia nigra and the globus pallidus.

## Teaser

Liu *et al.* analysed three large longitudinal Huntington’s disease imaging datasets using a single algorithm. They find dramatic selective atrophy of regions involved in the basal ganglia circuit. These results support the hypothesis of circuit-based spread of pathology in Huntington’s disease and have implications for current neurosurgical therapeutic approaches.

## Graphical Abstract

**Graphical Abstract:**
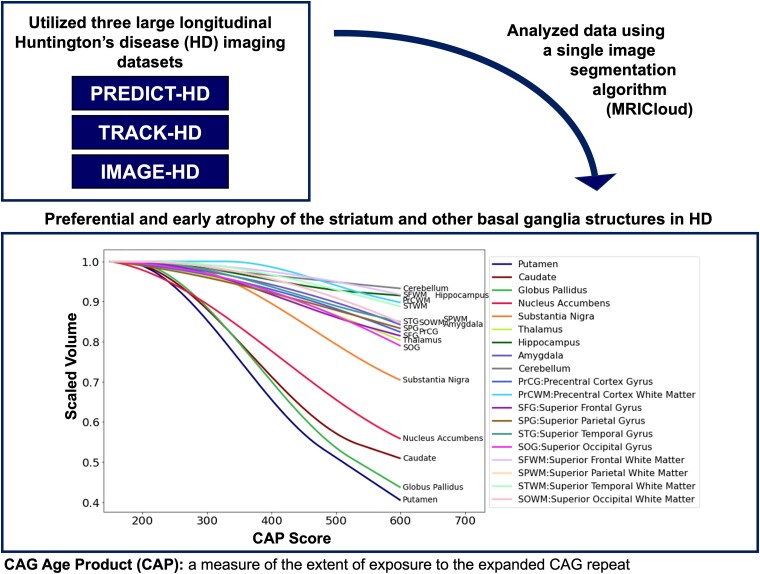

## Introduction

Huntington’s disease (HD) is caused by a CAG repeat expansion in the *Huntingtin* gene (*HTT*) on chromosome 4, which codes for polyglutamine in the Huntingtin protein (Htt). HD classically manifests with a triad of signs and symptoms, including motor, cognitive and behavioural features.^1,2^ Diagnosis is traditionally made based on clinician assessment of motor signs using the unified Huntington’s disease rating scale (UHDRS),^3^ though recent developments have added cognitive changes as contributing to the diagnosis of manifest HD.^4^

The age of onset of HD is strongly influenced by the length of the CAG trinucleotide expansion within the *Huntingtin* (*HTT*) gene. Above a threshold of around 36–39 CAG, the longer the repeat length, the earlier the onset. To estimate the effects of the CAG repeat expansion over time, a variable of the form Age×(CAG−L), where Age is the current age of the individual, CAG is the repeat length and *L* is a constant (reflecting an approximation of the threshold), was first proposed by Penny *et al*.^5^ It has subsequently been termed the ‘CAG Age Product’ (CAP) score.^1,6^ It enables the consideration of many individuals with the CAG repeat expansion at the same time for analysis of any variable and graphing using the CAP score as the X axis.

Huntington’s disease has traditionally been noted for selective striatal neurodegeneration, especially early in the course. The widely used Vonsattel neuropathological staging system focuses almost entirely on striatal atrophy and cell loss.^7^ However, there has been increasing appreciation that many other regions of the brain are affected, and in a recent review, HD was described as a ‘multisystem neurodegenerative disease’.^8^

Structural MRI studies have made possible great advances in our understanding of the natural history of neurodegeneration in HD. Initial single-site studies^9^ showed that progressive striatal atrophy begins many years prior to the onset of diagnosable motor HD. This has been amply confirmed by several large studies, including the PREDICT-HD and TRACK-HD multicentre studies, in North America and Europe, respectively, and the IMAGE-HD study in Australia.^10–14^ These studies, in addition to confirming the progressive early striatal atrophy, also called attention to atrophy in other areas such as subcortical white matter, for instance, as highlighted in the PREDICT-HD study. However, these previous studies, while very enlightening, have used different analytic methods and have emphasized slightly different results.

The present study has had the opportunity to analyse all three datasets together using a single image segmentation algorithm^15^ for all the scans. This has made it possible to combine data from a large number of subjects across a range of CAP scores. In addition, we have used a strategy of normalizing regional atrophy to atrophy of the whole brain, in order to determine which regions may undergo preferential degeneration. We demonstrate dramatic selective atrophy to regions involved in the basal ganglia circuit, such as caudate, putamen, nucleus accumbens, globus pallidus and substantia nigra. Most other regions of the brain appear to have slower but steady degeneration. These results have implications for the possibility of circuit-based spread of pathology in HD, suggesting pathophysiological mechanisms which may yield novel therapeutic targets. They also have implications for the design of neurosurgical approaches.

## Materials and methods

### Dataset

The data were obtained via CHDI from the previously published studies PREDICT-HD, TRACK-HD and IMAGE-HD.^10–12^ Each study was a longitudinal observational study including structural imaging. For PREDICT-HD, all HD subjects were premanifest at entry. TRACK-HD and IMAGE-HD had a mixture of premanifest and manifest subjects at entry. A similar approach as in this study was used in Wijeratne *et al.*,^16^ though with a different imaging pipeline, different analyses and information regarding the individual studies is also summarized there.

### Image preprocessing

All MRIs were automatically segmented via MRICloud,^15^ a public website platform for multi-contrast imaging segmentation and quantification. In MRICloud, the process of T1-WI segmentation, used for volumetric analysis, involves: (1) image pre-processing (orientation and inhomogeneity correction, skull stripping), (2) image registration to multiple atlases based on a sequence of affine transformations and the Large Deformation Diffeomorphic Mapping (LDDMM) and (3) multi-atlas labelling fusion (MALF) algorithm^17–19^ to fuse the parcellation labels of atlases for each scan. In this study, we used the atlas set ‘Adult50–90yrs_287Labels_30atlases_M2_252_V10A’, composed by 30 brain MRIs of 50–90 year-old individuals, in which 287 brain structures are defined with a multi-level hierarchical ontology.^20,21^ The performance of MRICloud pipeline, compared with human evaluators and with other state-of-the-art algorithms, was described in previous publications.^22–24^

The original images as well as the results of the brain segmentation from MRICloud were visually inspected for quality control (QC). Images were excluded in the presence of artefacts (e.g. too much motion) or in the case of incomplete coverage (e.g. scans did not cover the top or the bottom of the brain). We excluded cases with obvious registration errors, mostly derived from the very first step of the image processing, the linear mapping to the templates. These errors are well known in imaging processing and are usually related to large rotations in the interstice axis or particularities of the field-of-view. We opted not to perform any human correction on the segmentation, as our aim is to report the results of a consistent automated process across all the images. The sample sizes and participant demographic of all three datasets, before and after QC, are summarized in Table 1 in the Supplementary material. After QC, the percentage of male subjects in each study is 50%, 31.8% and 47% for IMAGE-HD, PREDICT-HD and TRACK-HD, respectively.

### Regions of interest

The regions of interest (ROI) considered for further analysis were the putamen, caudate, globus pallidus, nucleus accumbens, substantia nigra and thalamus, as well as the following cortical areas and white matter (WM) beneath them: precentral gyrus (PrCG, PrCWM), superior frontal gyrus (SFG, SFWM), superior parietal gyrus (SPG, SPWM), superior occipital gyrus (SOG, SOWM) and superior temporal gyrus (STG, STWM). The ROIs were selected for their consistent segmentation, as we attempted to analyse all regions. However, some regions of great interest such as the subthalamic nucleus and ventral diencephalon were not considered, as we found that their low contrast in T1-WIs of this dataset leads to less reliable segmentation. Brainstem was also not considered because the variable level of scan coverage could introduce artifactual variations. ROIs in both hemispheres were considered together as there is little evidence for significant asymmetry in HD.

### CAP score calculation

We aim to track the volume changes of each ROI over CAP scores among all three datasets (PREDICT-HD, TRACK-HD and IMAGE-HD). CAP scores were calculated across all three datasets by the following equation: CAP=Age×(CAG−33.66), where Age represents the age of subjects in years and CAG represents the (constant) length of the individual’s CAG repeat. A recent study^25^ has shown that MRI scans of far-from-onset HD subjects are, with one exception, essentially indistinguishable from controls, so we included controls under 40 years old to model very far from onset HD in this study. The exception is that there are small differences in striatal volumes, which were attributed to early degeneration or to subtle developmental differences. Modification of the CAP equation for controls is described in the Supplementary material. Analyses were conducted with this modification or with the CAP score set at CAP = Age.

### The regression model

In this study, we utilized a left-flat sigmoidal function and its statistical regression model, which are defined in detail in the Supplementary material, to capture the volume changes of each ROI over CAP scores. The rationale for this model is that most brain structure volumes in expansion-positive individuals far from onset are close to controls.^25^ Therefore, for CAG expansion-positive individuals very far from predicted onset, and for controls under 40, volumes can be modelled, as a first approximation, as constant. For all figures including controls, only controls under age 40 were used.

To reduce batch bias between datasets and subject groups, we considered datasets (PREDICT-HD, TRACK-HD and IMAGE-HD) and subject groups (i.e. controls, Pre-HD in Image-HD) as covariates in the statistical regression model. Subjects’ groups are defined according to the clinical criteria specified in their original dataset. We also included Intracranial Volume (ICV) as a covariate.

For a second set of analyses, the volume of each ROI was also normalized to the whole brain volume before statistical regression model fitting. The ICV corresponds to the volume inside skull, including CSF and brain parenchyma, while the whole brain volume includes only the volume of brain tissue, without CSF. Therefore, whereas ICV reflects the head size, whole brain volume is representative of the overall parenchymal atrophy. For changepoint analysis, we used a semi-parametric bootstrap method to calculate the *P*-values.

## Results

Table 1 shows the clinical characteristics of the subjects. For this plot, and all the others in the main figures, CAP score for controls is calculated as described in the supplemental text. (Additional details of the subjects and QC of the scans are shown in Tables 1 and 2A and B in the Supplementary material).

**Table 1:** Demographic, genetic and clinical characteristics of all participants at enrollment

	Controls (*n* = 178)^a^			HD (*n* = 357)^b^				
Variable	Mean	SD	Range	Mean	SD	Range	Χ^2^ (df)	*P*
Age (years)	45.2	11.0	22.6–71.9	43.6	11.3	18.6–71.6	2.0 (1)	0.161
CAP score^c^	252.9	61.6	126.6 to 402.7	377.9	89.6	149.6–631.6	200.4 (1)	<0.0001
CAG repeats	20.4	3.5	15 to 31	42.7	2.4	38–55	160.1 (1)	<0.0001
UHDRS-TMS	2.6	3.2	0 to 19	9.3	11.1	0–47	60.3 (1)	<0.0001
UHDRS-TFC	13.0	0.1	12 to 13	12.4	1.4	7–13	38.3 (1)	<0.0001
SDMT	53.8	9.6	28 to 80	46.9	13.3	12–80	35.1 (1)	<0.0001
BDI	4.8	5.3	0 to 22	6.4	7.2	0–43	1.7 (1)	0.195
Sex, male (%)	66 (37.1%)			149 (41.7%)			0.9 (1)	0.346
Study							0.3 (2)	0.881
IMAGE-HD	21 (11.8%)			42 (11.8%)				
PREDICT-HD	62 (34.8%)			132 (37.0%)				
TRACK-HD	95 (53.4%)			183 (51.3%)				

Comparison of HD and control participants in aggregate for the IMAGE-HD, PREDICT-HD and TRACK-HD studies. Only data from those participants whose imaging data (3 T only) passed the quality-control check are included. SD, standard deviation; Χ^2^, Chi-square; df, degrees of freedom; CAP, CAG-Age Product; UHDRS, unified Huntington’s disease rating scale; TMS, total motor score; TFC, total functional capacity; SDMT, symbol-digit modalities test and BDI, Beck depression inventory. Χ^2^ values and corresponding *P*-values are from the Kruskal–Wallis test for continuous variables and the Χ^2^ test for the nominal variable (sex).

a For control participants, CAG repeats were reported by PREDICT-HD only (*n* = 62), UHDRS by PREDICT-HD and TRACK-HD only (*n* = 157 for TMS and TFC), and BDI by PREDICT-HD and IMAGE-HD (*n* = 56).

b For HD participants, BDI was reported only by PREDICT-HD and IMAGE-HD (*n* = 122); UHDRS-TFC was reported only by PREDICT-HD and TRACK-HD (*n* = 312).

c Arbitrary value for CAP score for controls calculated as described in the text.

**Figure 1:**
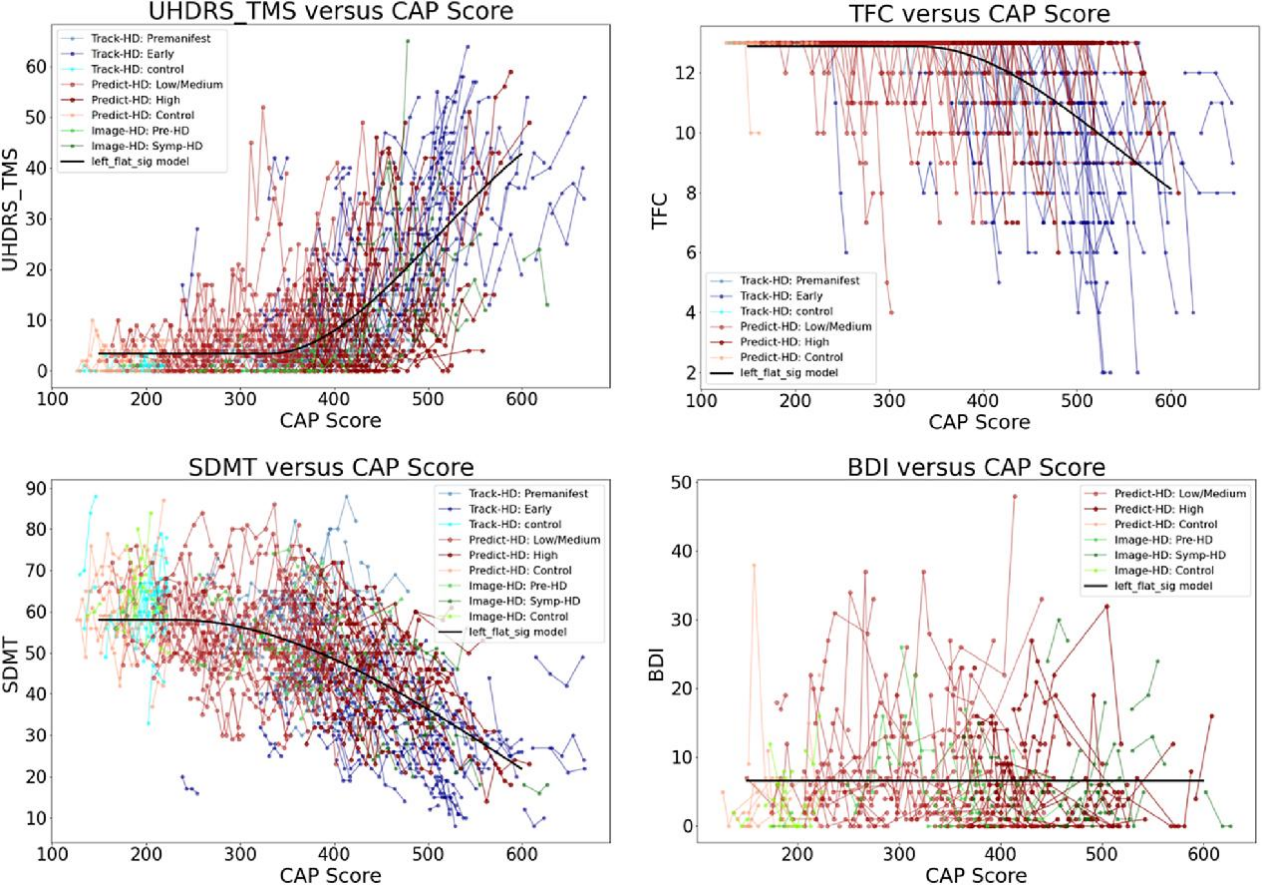
**Individual longitudinal clinical data (‘spaghetti plots’) from all three studies (PREDICT, TRACK and IMAGE).** TMS: total motor score; SDMT: symbol digit modalities test; TFC: total functional capacity; BDI: Beck depression inventory. Datasets appear in the following order: Track-HD: Premanifest, Track-HD: Early, Track-HD: control, Predict-HD: Low/Medium, Predict-HD: High, Predict-HD: Control, Image-HD: Pre-HD, Image-HD: Symp-HD, Left_flat_sig model. No available data for TFC from Image-HD and BDI from Track-HD.

Figure 1 shows the individual longitudinal clinical data (‘spaghetti plots’) of Total Motor Score (TMS), Symbol Digit Modalities Test (SDMT), Total Functional Capacity (TFC) and Beck Depression Inventory (BDI), where each of these were available, versus CAP score. As expected, inflection points in TMS and SDMT precede changes in TFC. Also, as expected, BDI (a reflection of depressive emotional state) shows no clear relationship to CAP score—emotional changes in HD do not occur with the predictability of motor or cognitive changes.

**Figure 2:**
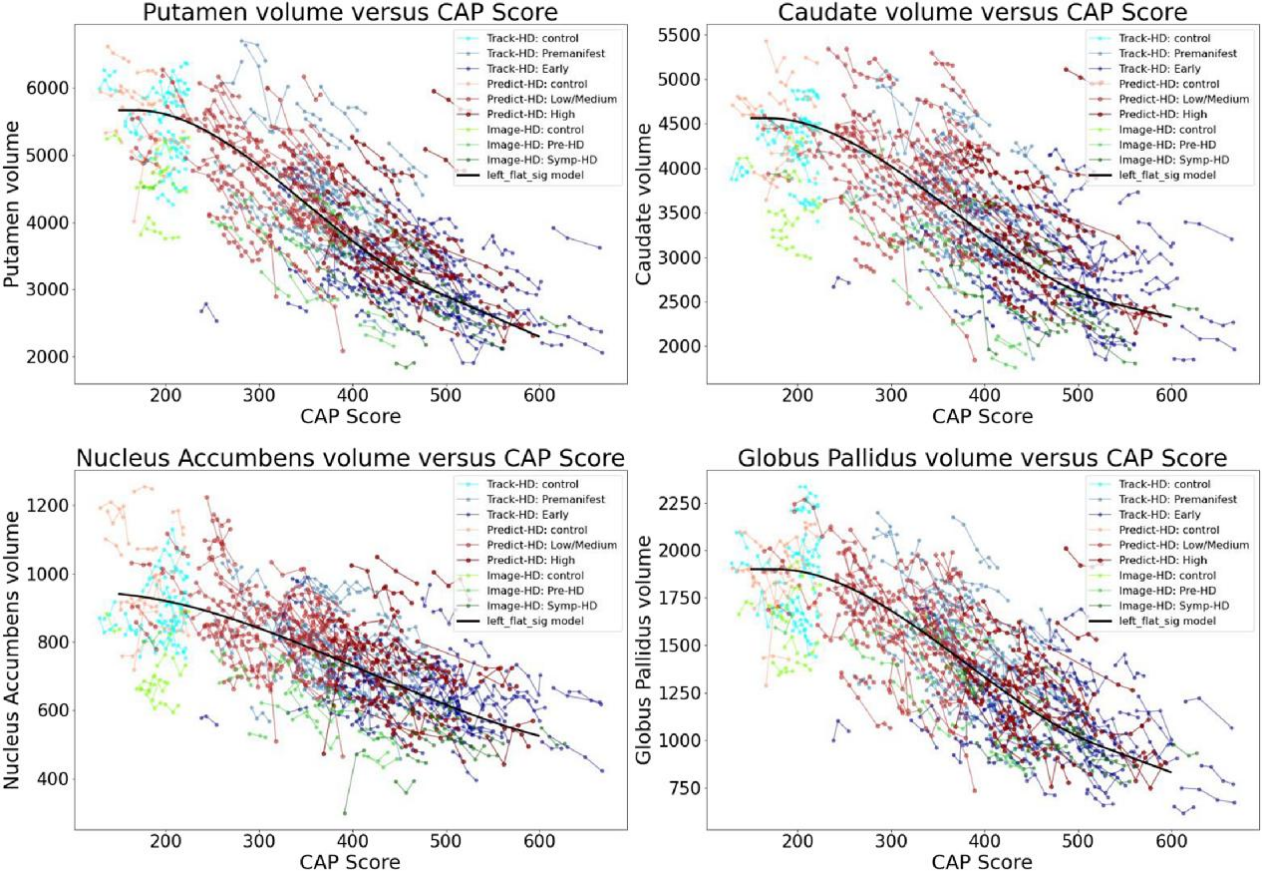
**Individual longitudinal volumetric data for selected regions (‘spaghetti plots’) from all three studies (PREDICT, TRACK and IMAGE), plotted by CAP score.** Covariate intracranial volume only (CAG expansion positive individuals and controls < age 40). Regions in the basal ganglia circuit have the most striking declines in volumes with greater CAP scores. Datasets appear in the following order: Track-HD: control, Track-HD: Premanifest, Track-HD: Early, Predict-HD: control, Predict-HD: Low/Medium, Predict-HD: High, Image-HD: control, Image-HD: Pre-HD, Image-HD: Symp-HD, Left_flat_sig model.

**Figure 3:**
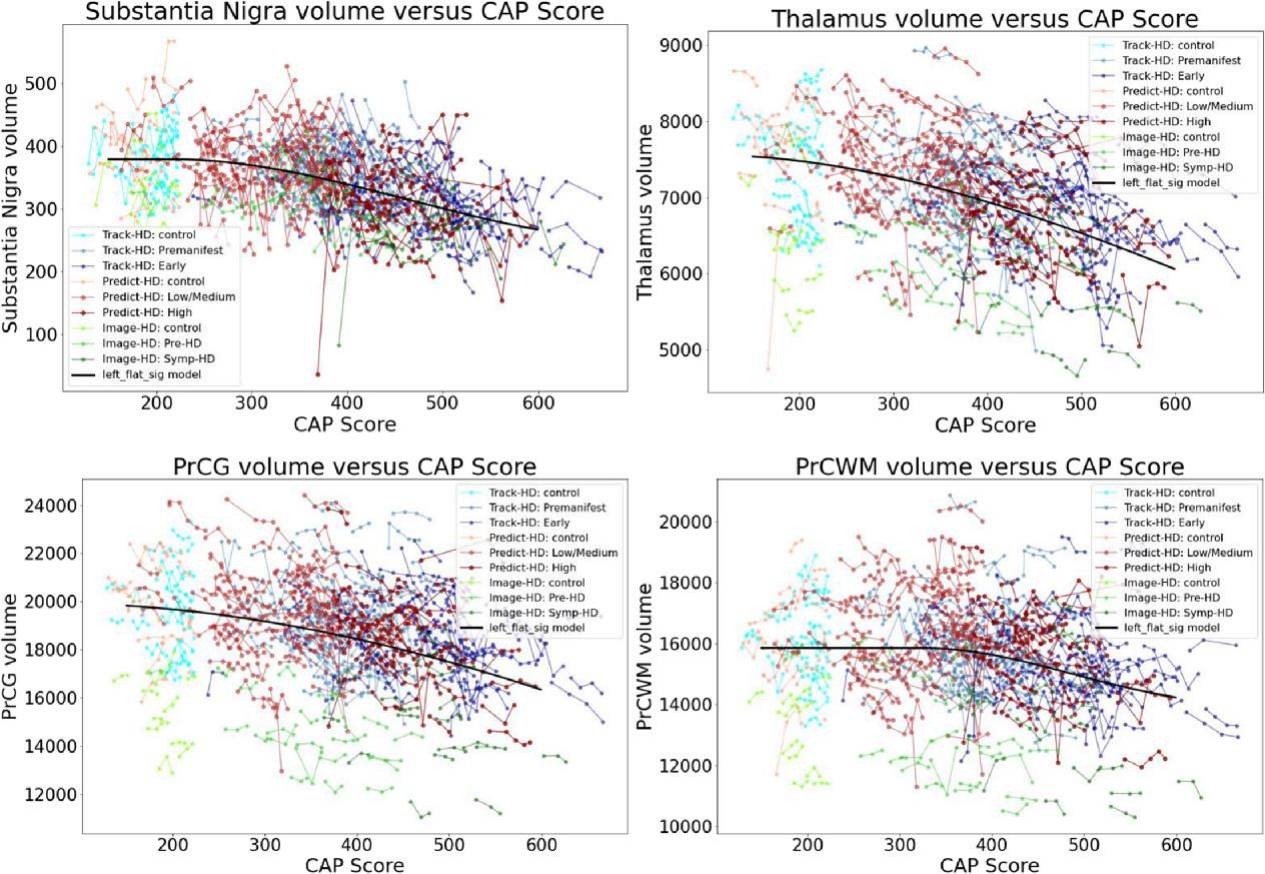
**Individual longitudinal volumetric data for selected regions (‘spaghetti plots’) from all three studies (PREDICT, TRACK and IMAGE), plotted by CAP score.** Covariate intracranial volume only (CAG expansion positive individuals and controls < age 40). Regions in the basal ganglia circuit have the most striking declines in volumes with greater CAP scores. Refer to Fig. 2 for complete dataset list.

Figures 2 and 3 show the individual longitudinal volumetric data for selected regions (‘spaghetti plots’) from all three studies, plotted against CAP score. Intracranial volume is one of the covariates (but whole brain volume is not). Regions in the basal ganglia circuit have the most striking declines in volumes with greater CAP scores.

**Figure 4:**
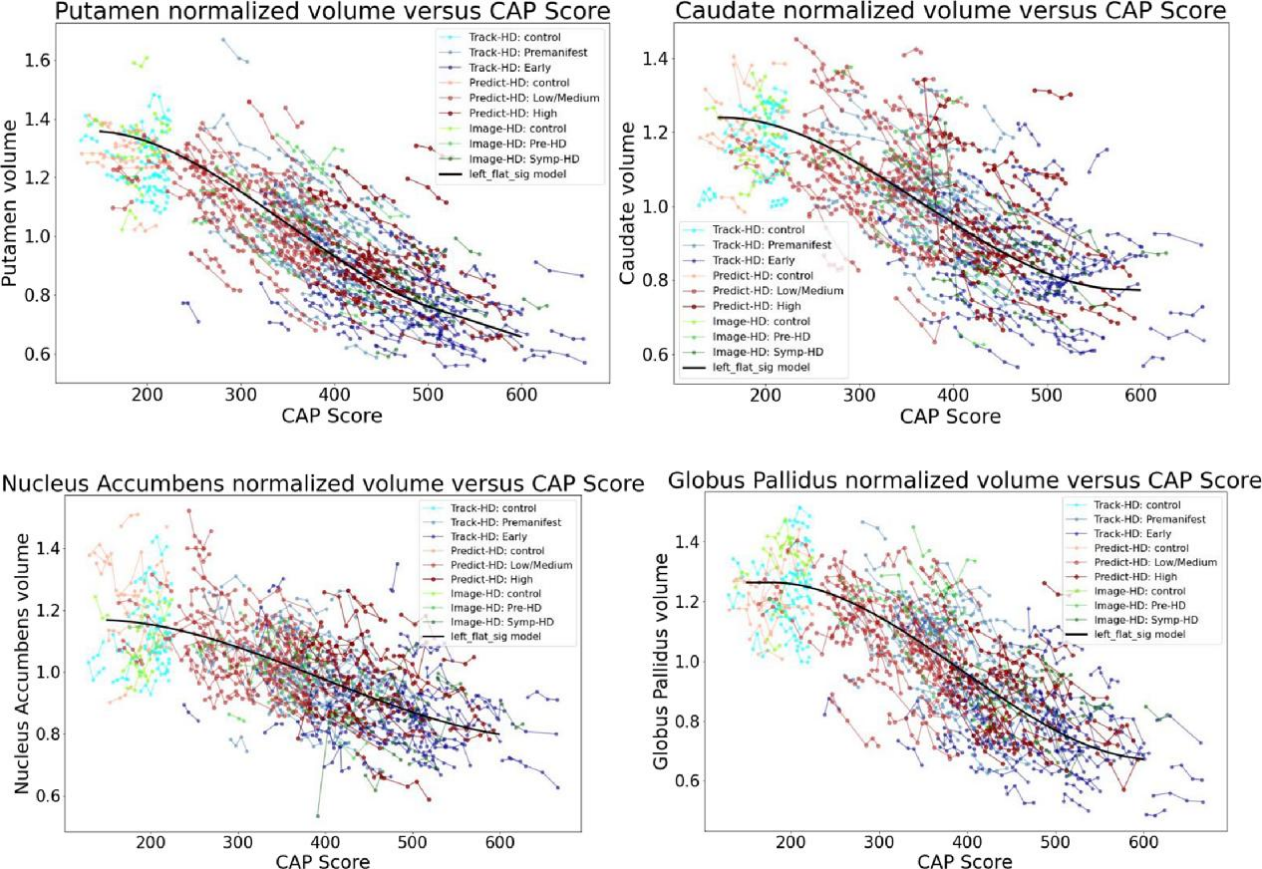
**Individual longitudinal volumetric data for selected regions (‘spaghetti plots’) from all three studies (PREDICT, TRACK and IMAGE), plotted by CAP score.** Covariate intracranial volume, plus normalization by whole brain volume (CAG expansion positive individuals and controls < age 40). This analysis highlights the extent to which regions in the basal ganglia circuit have the most striking declines in volumes with greater CAP scores. Refer to Fig. 2 for complete dataset list.

**Figure 5:**
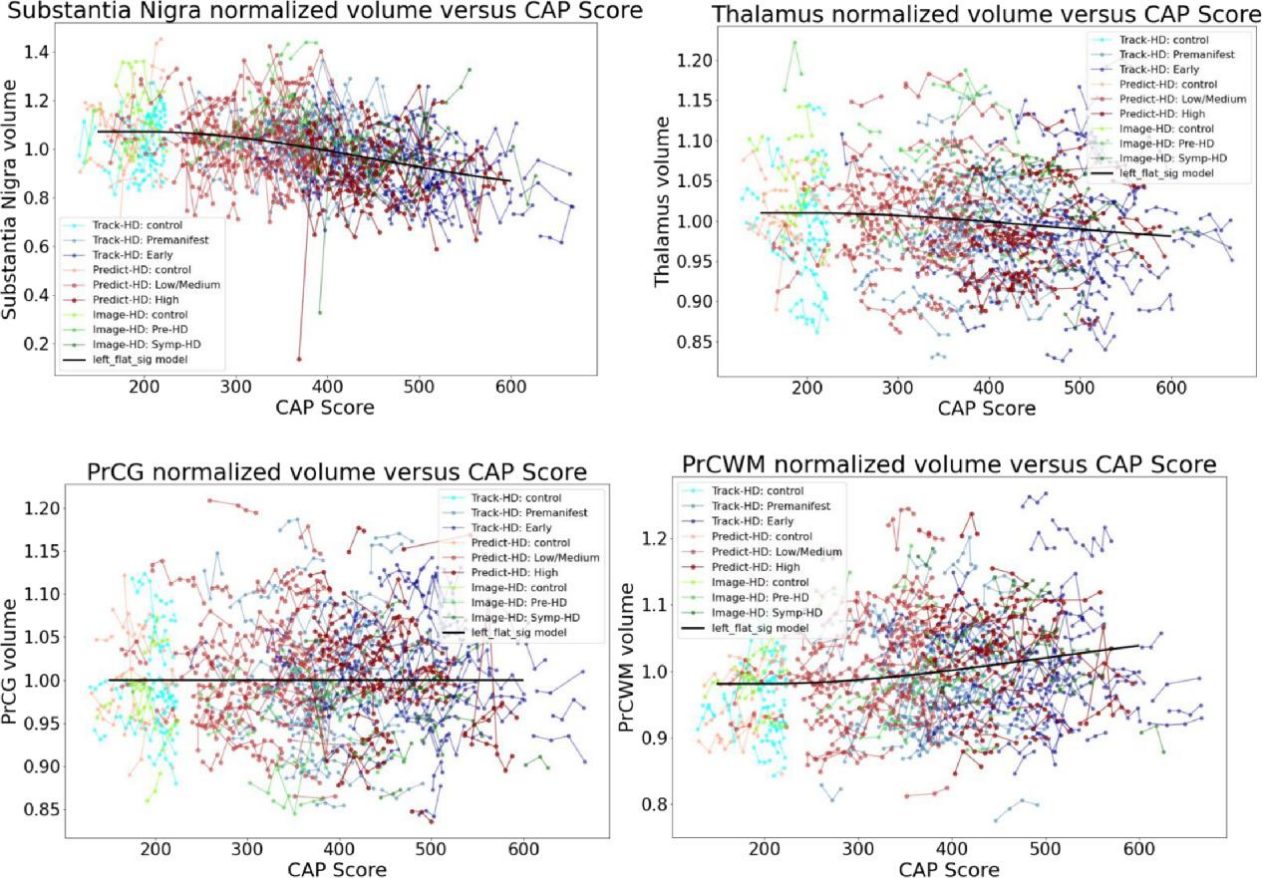
**Individual longitudinal volumetric data for selected regions (‘spaghetti plots’) from all three studies (PREDICT, TRACK and IMAGE), plotted by CAP score.** Covariate intracranial volume, plus normalization by whole brain volume (CAG expansion positive individuals and controls < age 40). This analysis highlights the extent to which regions in the basal ganglia circuit have the most striking declines in volumes with greater CAP scores. Refer to Fig. 2 for complete dataset list.

Figures 4 and 5 show individual longitudinal volumetric data for selected regions (‘spaghetti plots’) from all three studies, plotted against CAP score. Intracranial volume (ICV) is a covariate, as above, but here there is also normalization by whole brain volume. This analysis highlights the extent to which regions in the basal ganglia circuit have the most striking declines in volumes with greater CAP scores.

**Figure 6:**
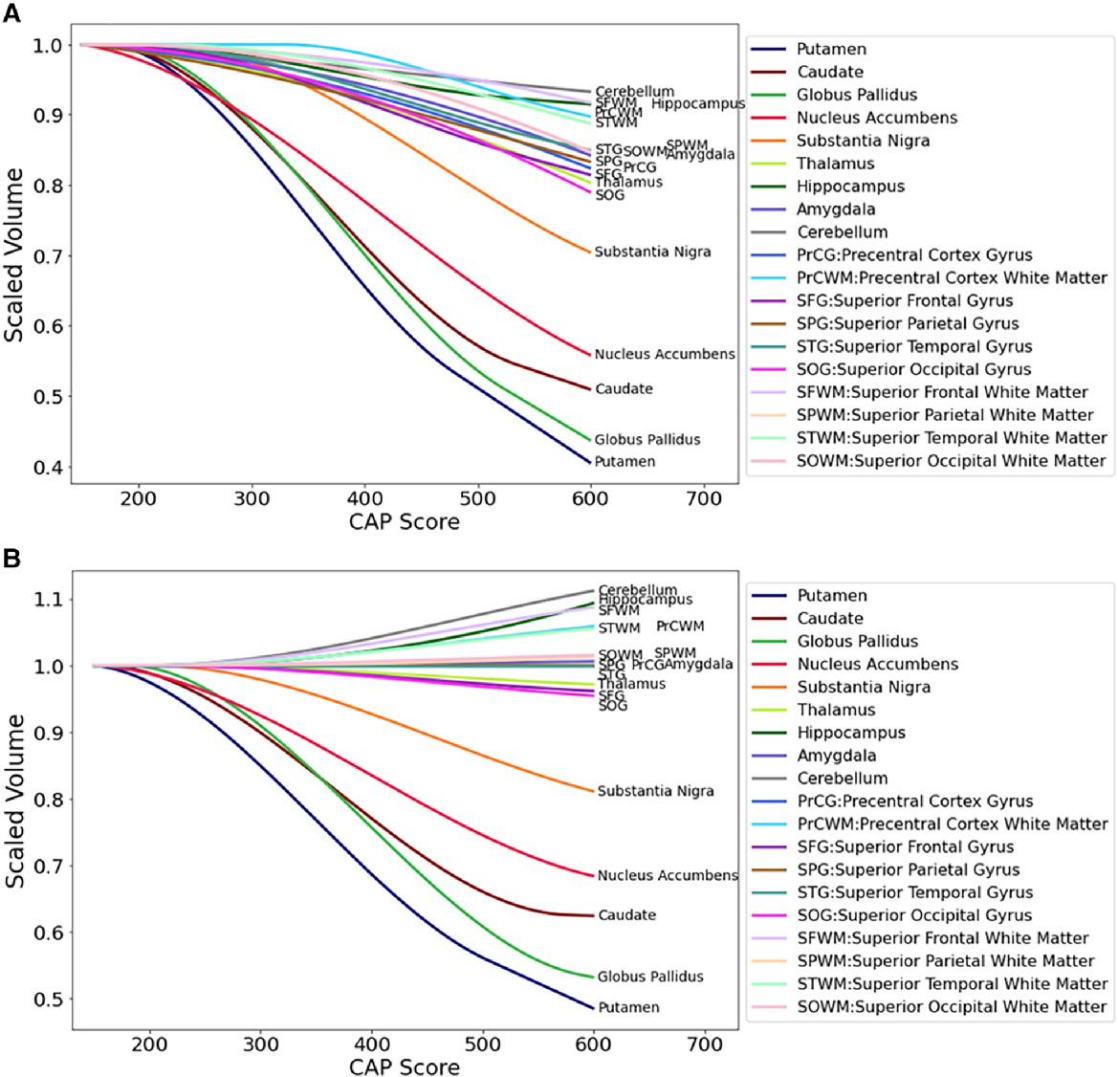
**Summary trend lines for selected regions.** (**A**) Covariate intracranial volume only (CAG expansion positive and controls < age 40). (**B**) Covariate intracranial volume and normalized by whole brain volume (CAG expansion positive and controls < age 40). These comparisons highlight the extent to which regions in the basal ganglia circuit have the most striking declines in volumes with greater CAP scores, especially when normalized by whole brain volumes. Note that regions whose relative volumes increase in Panel (**B**) do not have actual increase in volume—they just have less atrophy than the whole brain, i.e. they are relatively spared.

Figure 6 shows the summary trend lines for selected regions. **A**. Covariate intracranial volume only. **B**. Covariate intracranial volume plus normalization by whole brain volume. These comparisons highlight the extent to which regions in the basal ganglia circuit have the most striking declines in volumes with greater CAP scores, especially when normalized by whole brain volumes. Note that regions whose relative volumes increase in Panel **B** do not have actual increase in volume—they just have less atrophy than the whole brain, i.e. they are relatively spared.

Supplemental Fig. 1 shows additional brain regions. Supplemental Fig. 2 shows additional brain regions, normalized by whole brain volume. Supplemental Fig. 3 shows analyses with control CAP = Age. Supplemental Fig. 4 provides the CAP score at first slope change for the sigmoidal model applied to each volume, and its standard deviation. Only volumes for which the sigmoid fitting was better than a linear model are listed. **A** provides results for covariate intracranial volume only and **B** for normalized by whole brain volume. Results in both cases indicate an earlier changepoint for putamen and caudate, followed by a group of structures that include the globus pallidus, the nucleus accumbens and the white matter in the precentral cortex. Several of the changes detected in un-normalized volumes lose their significance after normalization, suggesting that these changes are in part due to global brain volume loss.

**Figure 7:**
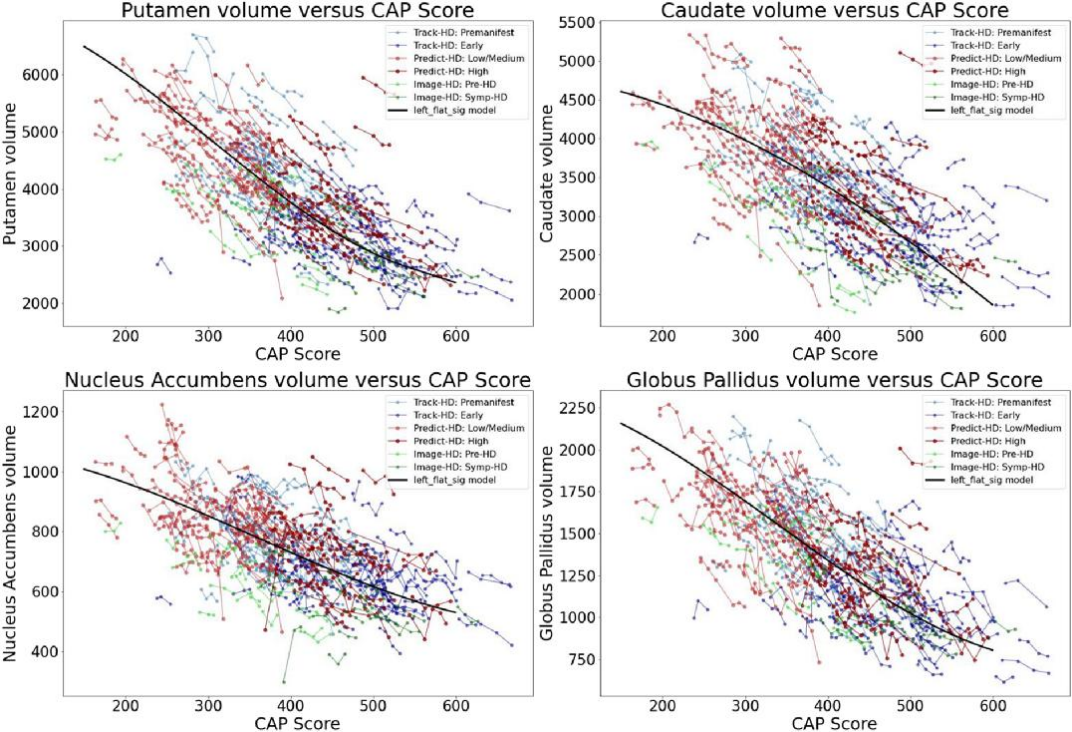
**Individual longitudinal volumetric data (‘spaghetti plot’) for selected regions.** Covariate intracranial volume only (CAG expansion positive only, no controls). Datasets appear in the following order: Track-HD: Premanifest, Track-HD: Early, Predict-HD: Low/Medium, Predict-HD: High, Image-HD: Pre-HD, Image-HD: Symp-HD, Left_flat_sig model.

**Figure 8:**
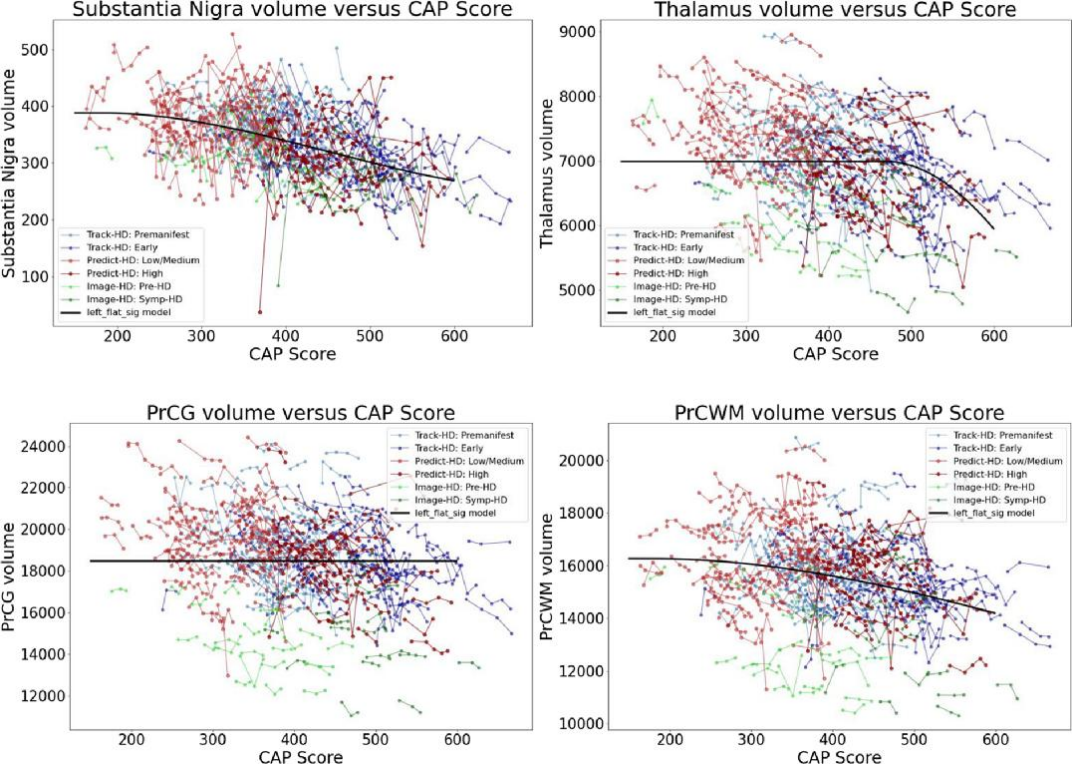
**Individual longitudinal volumetric data (‘spaghetti plot’) for selected regions.** Covariate intracranial volume only (CAG expansion positive only, no controls). Refer to Fig. 7 for complete dataset list.

**Figure 9:**
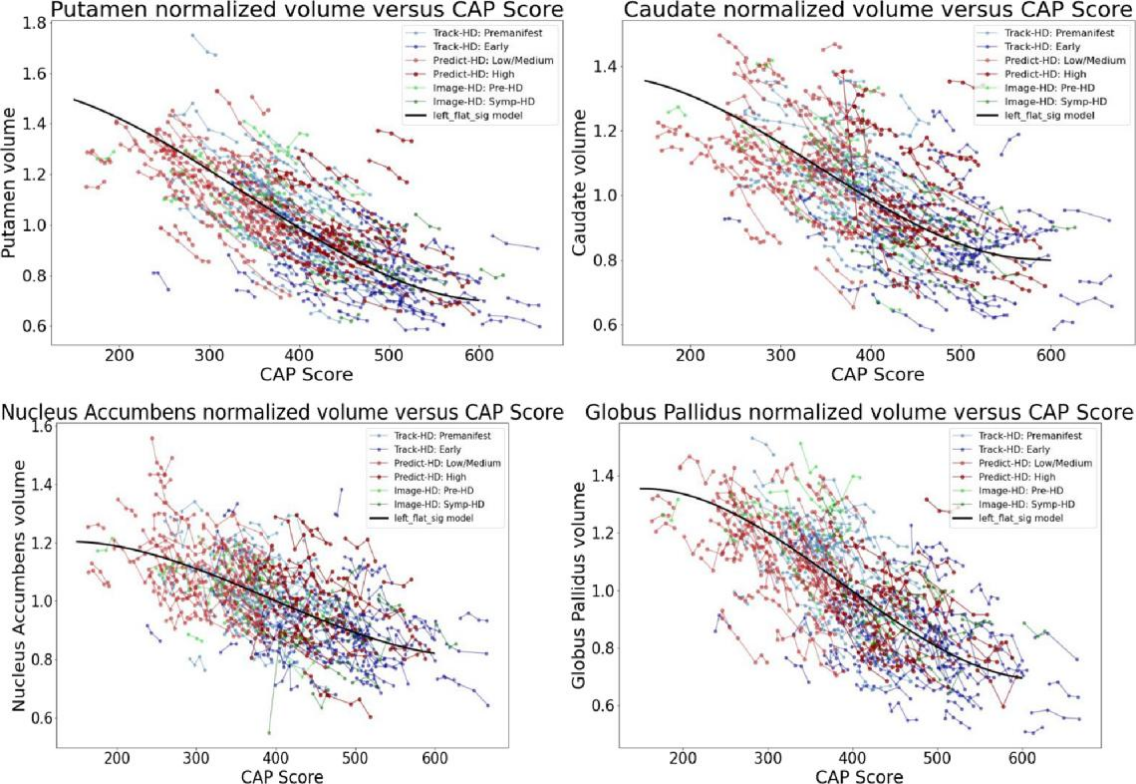
**Individual longitudinal volumetric data (‘spaghetti plot’) for selected regions.** Covariate intracranial volume, plus normalization by whole brain volume (CAG expansion positive only, no controls). Refer to Fig. 7 for complete dataset list.

**Figure 10:**
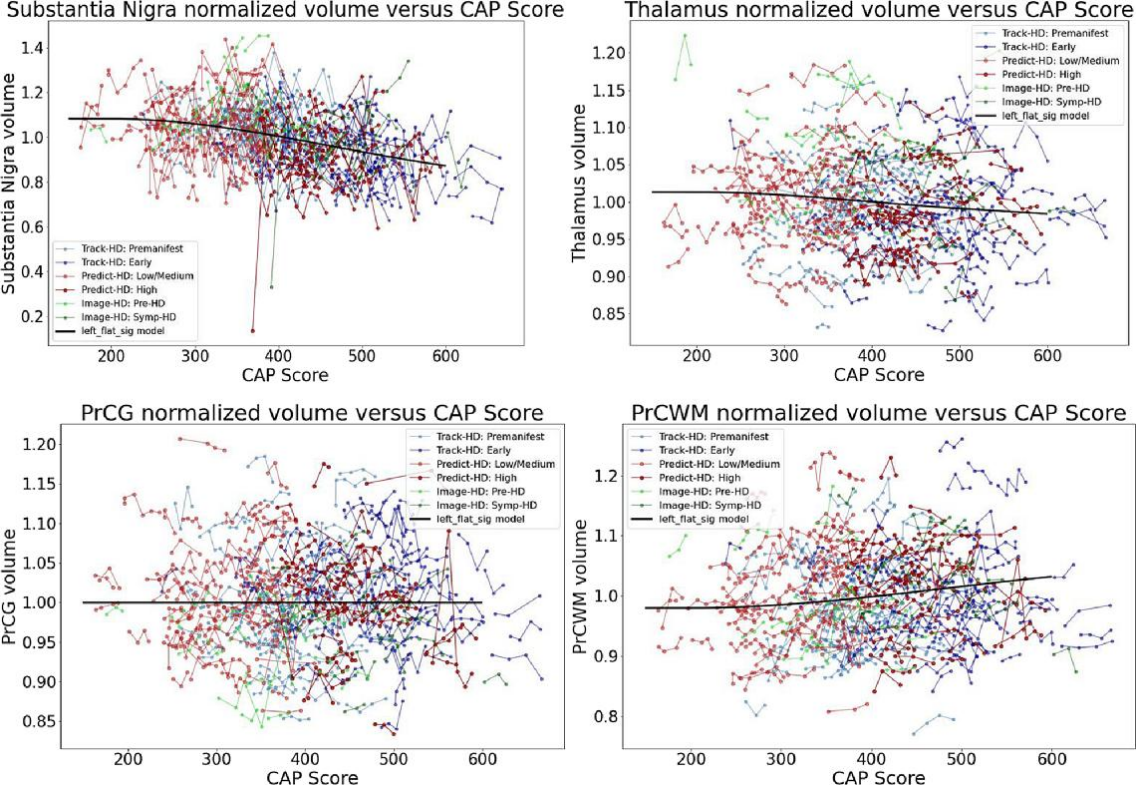
**Individual longitudinal volumetric data (‘spaghetti plot’) for selected regions.** Covariate intracranial volume, plus normalization by whole brain volume (CAG expansion positive only, no controls). Refer to Fig. 7 for complete dataset list.

**Figure 11:**
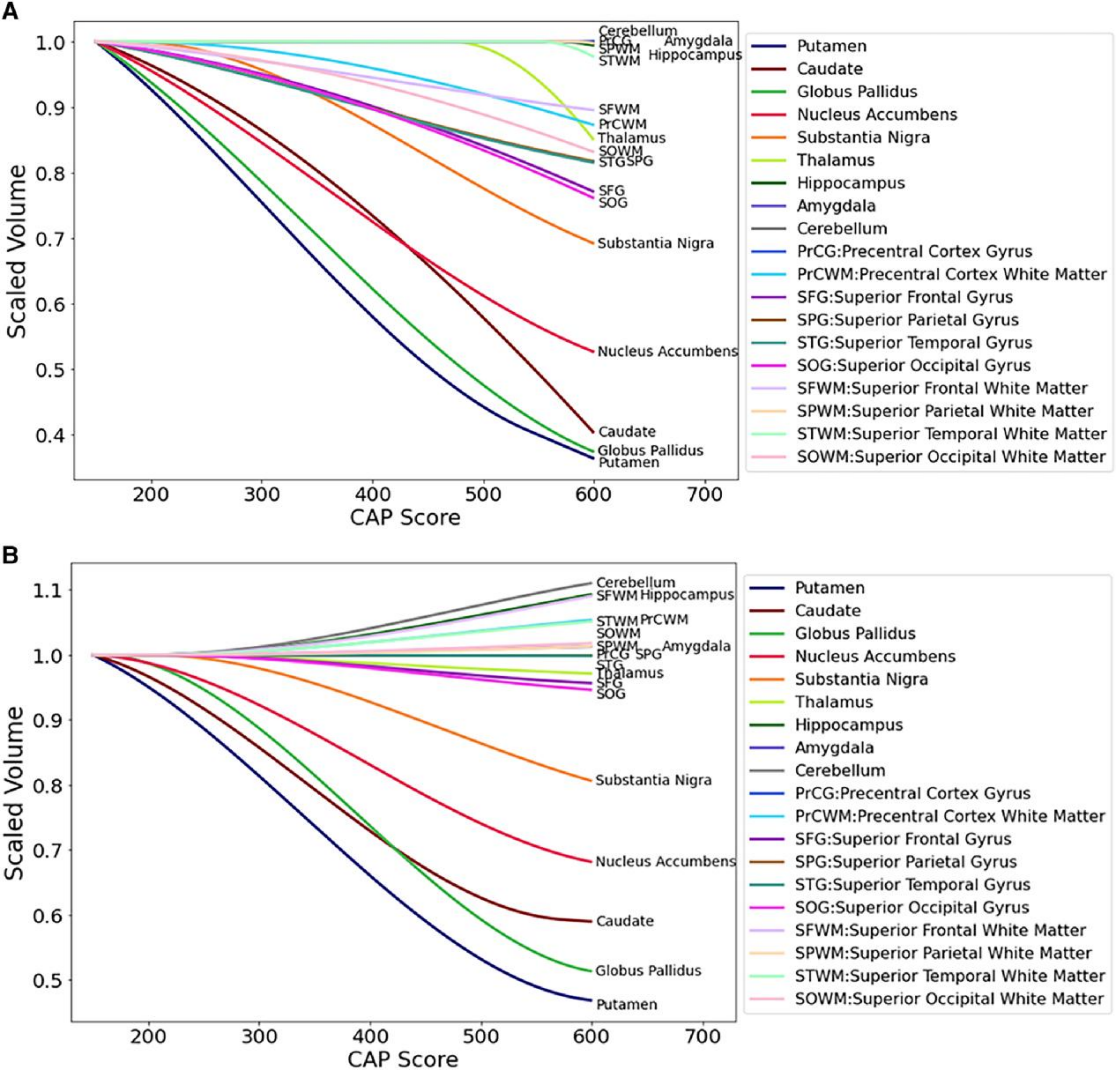
**Summary trend lines for selected regions, CAG expansion positive only, no controls.** (**A**) Covariate intracranial volume only. (**B**) Covariate intracranial volume, plus normalization by whole brain volume. These analyses also indicate preferential atrophy of the structures in the basal ganglia circuit.

Since analyses by CAP scores including controls require somewhat arbitrary methods to assign controls a CAP score, we also conducted analyses using HD cases only. Figures 7–10 show similar analyses with CAG expansion cases only, no controls. Supplemental Figs 5 and 6 show analyses of additional brain regions. Figure 11 shows trend lines for CAG expansion subjects only. These analyses also indicate preferential atrophy of the structures in the basal ganglia circuit.

## Discussion

These data highlight the selective vulnerability to progressive atrophy in HD of subcortical regions in the basal ganglia circuit. They raise the interesting possibility that there are circuit-based processes underlying regional brain atrophy in HD. As expected, the striatum has profound selective, early degeneration. Other regions of the brain within the striatal circuit also have selective severe degeneration. The globus pallidus in particular has severity of degeneration comparable to that of the striatum. In addition, regions in the basal ganglia circuit such as the nucleus accumbens and substantia nigra also have preferential atrophy. By contrast, most of the other brain regions examined appear to undergo slow but steady atrophy within a narrower range, with some being relatively spared.

A great strength of the study is the analysis of the large number of structural MRI images using the same analytic pipeline.^15^ This permits the combining of different datasets, and enhances the generalizability of the results. To our knowledge, this study represents the most comprehensive delineation of the natural history of regional brain atrophy in HD to date, combining TRACK, PREDICT and IMAGE participants. We encapsulated an extensive list of brain regions and showed individual trend lines to underscore selective alteration of the basal ganglia circuitry during disease progression. We also identified the time course of slope change for regions of interest, which highlights early striatal degeneration.

Another great strength is that the combination of the three datasets includes subjects with a wide range of CAP scores. Most subjects were relatively early in the course of HD, i.e. in the premanifest period, though some started in the manifest period, and some ‘converted’ to motor manifest HD^26^ during the course of the study. Diagnosis of HD in these studies was made using clinician assessment of motor signs using the unified Huntington’s disease rating scale (UHDRS),^3^ not the more recent incorporation of cognitive changes.^4^ Thus, overall there is a range of subject CAP scores, covering well the key period of early to moderate brain atrophy, which would not have been available studying any single dataset.

However, there are a number of limitations which must be acknowledged. The automated analysis platform,^15^ while highly sophisticated and capable of dealing with selective regional brain atrophy due to the use of the LDDMM method, does not have the face validity of expert manual segmentation for each individual region. However, it would be impossible to analyse so many scans without automated image analysis. As described in the methods, all scans did have manual quality control. Another segmentation issue, faced by both humans and computational methods, is that small regions, and those with relatively low contrast, may be especially liable to inconsistent results. Therefore, the results must be interpreted in the light of the regional segmentation reliability.^24^ There are some important regions which were too small or too uncertain to segment and were not included, such as the brain stem, subthalamic nucleus and ventral diencephalon. There is little evidence of asymmetric atrophy is HD, so our study did not analyse the two hemispheres separately, though this could be explored in the future if felt to be desirable. Furthermore, our results are limited to volumetric analysis. Other methods such as voxel-based morphometry, or other morphometry-based techniques might give complementary results.^27–29^

Other limitations involve the range of CAP scores. Our trend lines are limited to the range of CAP scores between 200 and 600, because we do not have sufficient data outside of that range. These studies did not include images from HD individuals with severe disease, who are often difficult to scan because of involuntary movements or other clinical issues. In addition, there is a lack of individuals very far from predicted onset.

Because of the recent important study from Scahill *et al.*,^25^ we know that individuals very far from onset have most regional volumes essentially equivalent to normal, with the exception of slight but significant differences in striatal volumes; therefore, we have used the expedient introduction of normal controls into the analysis in order to overcome this limitation. The slightly different striatal volumes in the very far-from-onset individuals^25^ could be due to atrophy beginning even earlier in the natural history of HD or due to a contribution from developmental differences in HD^30–33^ or possibly both. If the former is correct, then the use of controls as surrogates for very far-from-onset subjects will give a good overall description of the extent of atrophy. If the latter is correct, then our striatal data should be interpreted as reflecting a combination of a small component of developmental differences, plus the major component of degeneration. Because of the lack of a large number of subjects with CAP less than 200, it is difficult to define the CAP scores at which atrophy begins, so we do not attempt to draw conclusions here about the initiation of atrophy in different regions.

The use of the multiplier to assign the control subjects the equivalent of a CAP score is arbitrary. However, it helps to spread their data out over a larger range of scores. The goal of the study was to compare the relationship of atrophy of different brain regions in the HD subjects with CAP scores, and the use of the controls to substitute for very far from onset subjects will apply to all brain regions equally. We show that the overall patterns of results are very comparable using control CAP = Age (supplemental Fig. 3) or analysing the CAG expansion subjects only (Figs 5–7) without inclusion of controls.

Our results are broadly similar to those of Wijeratne *et al.*,^16^ though we have emphasized longitudinal trajectory (including with normalization for total brain volume), rather than effect sizes and markers and power for clinical trials. Technical differences include the analytic pipelines used, and the inclusion of the 1.5 T scans from PREDICT in their study, but not in the current study. Another study of Wijeratne *et al.*^34^ detected early changes in subcortical regions of the striatum using the Gaussian process progression model but with only TRACK and PREDICT participants, whereas we included the IMAGE study and a more thorough list of brain regions to demonstrate evidence of circuitry-based atrophy. They confirmed that structural brain atrophy provides improved prediction of onset of manifest HD, as previously shown in the PREDICT dataset alone.^26^ While they state that ‘monotonicity in the group-level volumetric evolution was enforced,’ nevertheless, their model, which involves many degrees of freedom, has the curves changing direction (for instance, caudate turns up towards the end, while lateral ventricle turns down), which does not fit the biology of HD. We believe that the sigmoid function that we used provides a better reflection of the progressive monotonic but non-linear nature of neurodegeneration in HD, and we show that it provides a statistically superior fit to the data compared to a linear model. Our results are also congruent with those of Abeyasinghe *et al.*,^35^ though their focus on describing trajectories (using Freesurfer) for subjects with different CAP scores was very different from the current one. They found that striatal volumes alone captured much of the variance in disease progression expressed by clinical variables, highlighting the importance of imaging as a potential outcome measure for clinical trials. It would be interesting to compare the results of the analyses in this study with similar analyses of Freesurfer data or other analytic pipelines.

Most regions of the brain, including many regions shown in the supplementary data, appear to undergo slow but steady atrophy over the longitudinal course of HD. By contrast, the striatum and other regions in the basal ganglia circuit undergo preferentially severe degeneration. This suggests the possibility of two mechanisms underlying neuronal degeneration in HD—perhaps all regions of the brain are affected by cell-autonomous mechanisms with roughly equivalent rates of atrophy, including white matter regions.^36^ This would be consistent with many animal model studies showing that not just neurons but other cells can contribute to HD pathogenesis. The cerebellum is sometimes thought of as a control region relatively unaffected by HD. However, our data suggest that cerebellum, while one of the relatively less affected regions, is within the broad group of regions undergoing slow but steady atrophy, and for instance is quite comparable to the hippocampus.

While these regions have relatively steady atrophy, there is substantial variation from case to case, so there are likely to be many opportunities for more detailed study of individual regions, and for clinical correlations, as have been done in the individual datasets. For instance, regional cortical atrophy has been shown in each dataset individually.^12,37,38^ For the cortex, thickness may be more revealing than volumetric analysis, but even in the current study using cortical volumes, there appear to be differences in different regions. Newer methods of analysing cortical thickness^39^ may be useful for clinical correlations. Also, subcortical regions may have interesting correlations. For instance, amygdala atrophy was correlated with depression in the IMAGE dataset.^40^ This finding suggests different HD phenotypes may be associated with alterations of specific structures, apparent at various timepoints throughout disease progression, not all of which are revealed in our study.

By contrast the striatum and other regions within the basal ganglia circuit undergo preferential and more profound degeneration. The basal ganglia selectivity raises the possibility of pathogenesis dependent on neuronal circuits, as has been suggested previously.^1,41–44^ Excitotoxicity has long been believed to contribute to HD pathogenesis in the striatum.^45^ There is considerable evidence for excitotoxic injury in HD.^46^ The severe involvement of the globus pallidus would appear inconsistent with excitotoxicity. The projection from the cortex to the striatum provides massive glutamatergic input to medium spiny neurons, and thus could be a substrate for excitotoxicity. However, the projection from the stratum to the globus pallidus is purely comprised of inhibitory neurons using GABA as a transmitter. Thus, excitotoxicity would not be possible for this synaptic interaction, though it is conceivable that glutaminergic innervation from the subthalamic nucleus^47^ could contribute. It must be acknowledged that the pallidum has relatively few neurons compared to the striatum,^48^ so it may be that atrophy there results from massive loss of afferent terminals as well as neuronal loss.^49^ White matter pathology has been well established in HD.^50–54^ The thalamus receives basal ganglia projections, but is not preferentially affected; however, it has many nuclei that do not receive basal ganglia projections, so a subnuclear or shape analysis may be more revealing.

Another mechanism might involve prion-like transmission of mutant Htt from one neuron to another. Network-based preferential atrophy and the mechanism of prion-like transmission have been proposed in Parkinson’s disease and Alzheimer’s disease,^28,55,56^ as well as of course in prion disease,^57,58^ though it may not be the only explanation for circuit-based atrophy. There are some striking data that favour this mechanism for HD as well, including in-vitro and in-vivo experiments, and observation of human postmortem brain material from transplant studies.^59–65^ Other potential mechanisms include loss of growth factor transport, or altered synaptic connectivity,^1,66–68^ perhaps due to abnormal complement-mediated pruning.^69^ The recent identification of modifier genes involved in DNA repair has led to hypotheses based on somatic expansion of the CAG repeat.^70,71^ The regional variation in somatic expansions^72^ appears to match the regional variation in extent of atrophy; though more detailed studies of this relationship could be revealing. Thus, the apparent circuit-based preferential pathology could have several explanations, as has also been proposed for PD.^73^ Furthermore, other mechanisms, such as cell-autonomous effects, must explain the overall atrophy present in later stage cases of HD, especially in juvenile onset HD.^74^

In conclusion, these data provide a comprehensive description of the natural history of regional volumetric brain atrophy in HD. They highlight the widespread degeneration of many (and perhaps all) regions of brain over the course of HD. They also highlight the selective atrophy of regions in the basal ganglia circuit, with implications for pathogenesis and experimental therapeutics. These results support (though certainly do not prove) the hypothesis of circuit-based spread of pathology in HD, possibly due to spread of mutant Htt protein, supporting therapeutic targets related to prion-like spread of pathology or other connection-based mechanisms. In addition, they have implications for current neurosurgical therapeutic approaches, since delivery of therapeutic agents solely to the caudate and putamen may miss structures affected early, such as nucleus accumbens, and output nuclei of the striatum, the substantia nigra and globus pallidus.

## Supplementary Material

fcad214_Supplementary_Data

## Data Availability

The study is ongoing with the images still under analysis. The individual brain regional volumetric data reported here will be provided to qualified investigators upon request and discussion.
