# Supplementary material for: Longitudinal imaging highlights preferential basal ganglia circuit atrophy in Huntington’s disease
Source: Brain Commun. 2023 Aug 18;5(5):fcad214. doi: 10.1093/braincomms/fcad214 (PMC10516592; doi:10.1093/braincomms/fcad214)
Supplement: fcad214_Supplementary_Data [file fcad214_supplementary_data.zip › Supplementary_figures.docx]

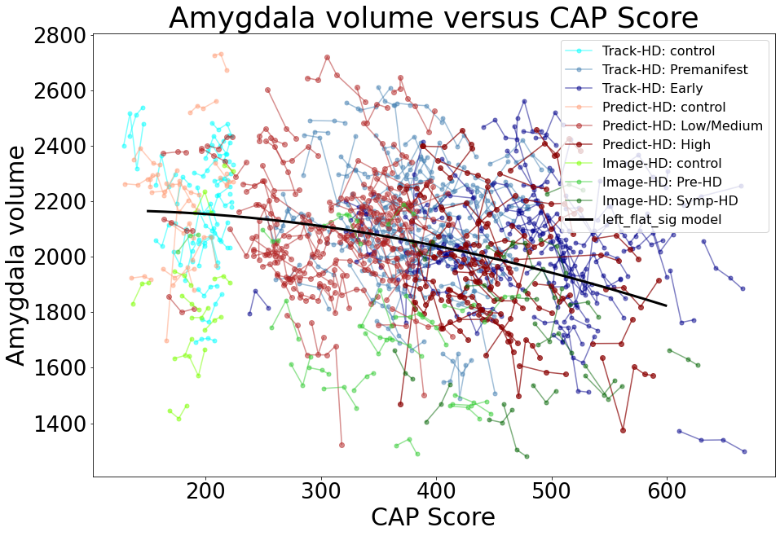

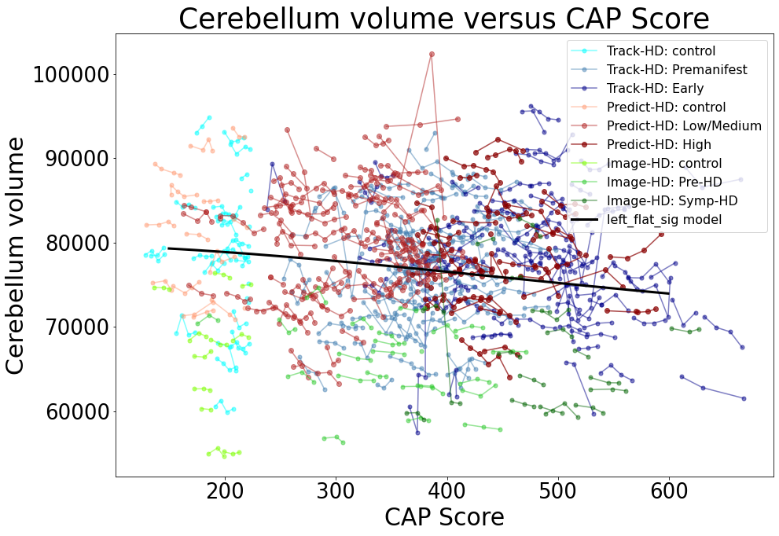

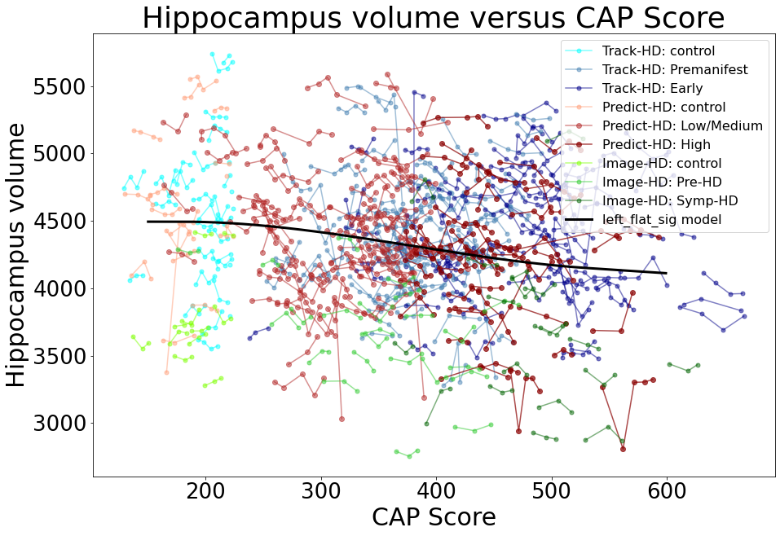

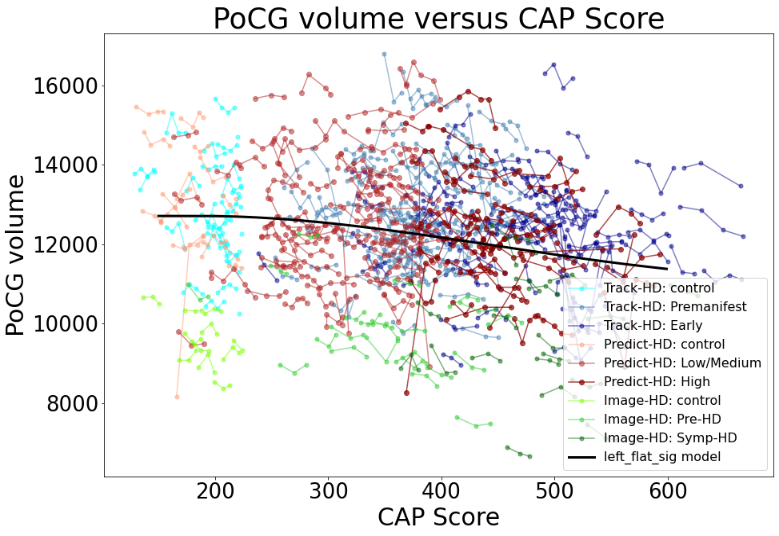

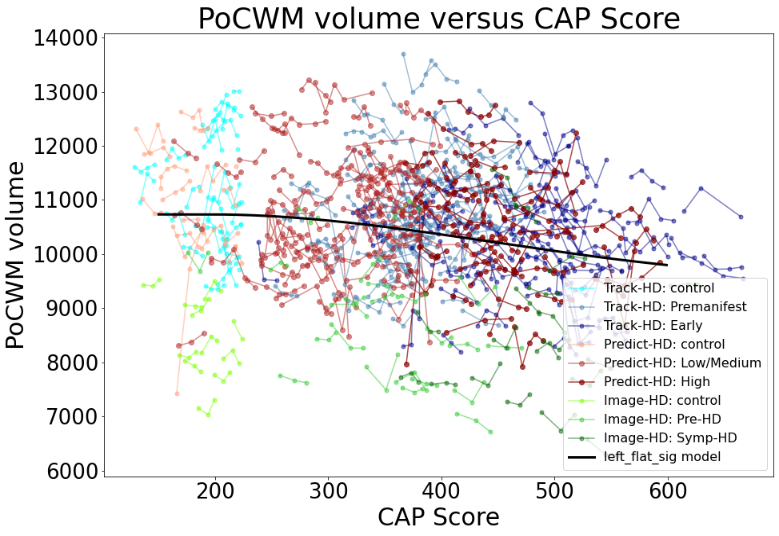

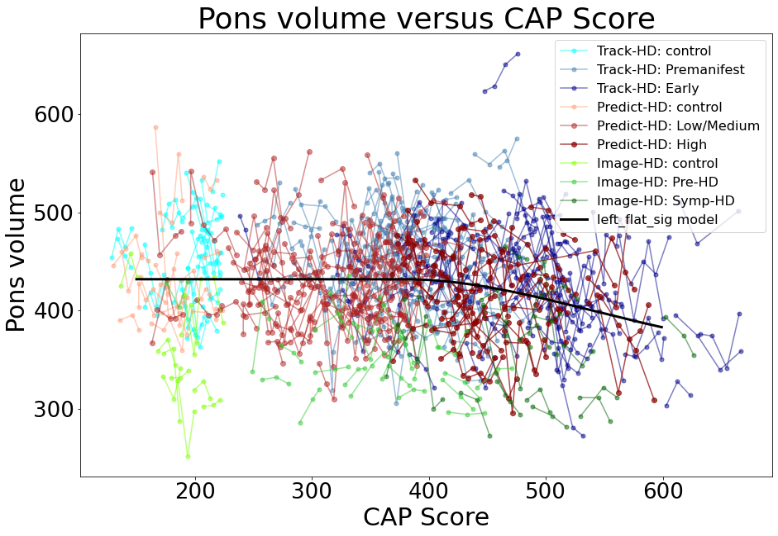

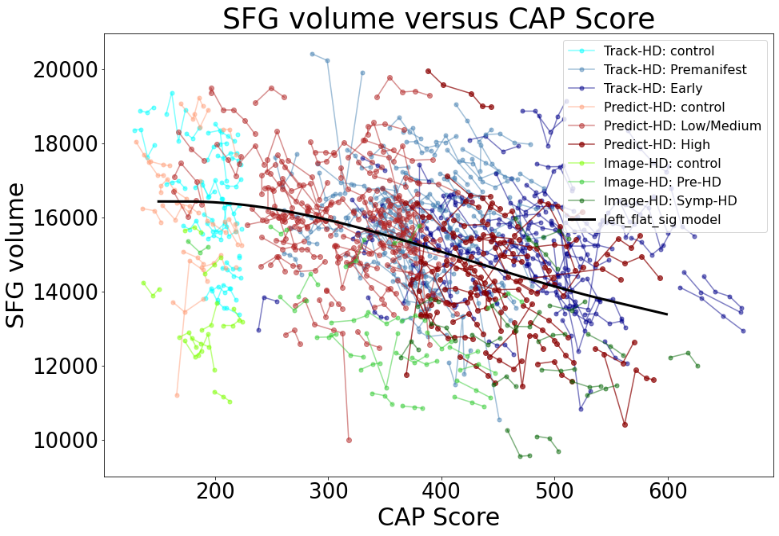

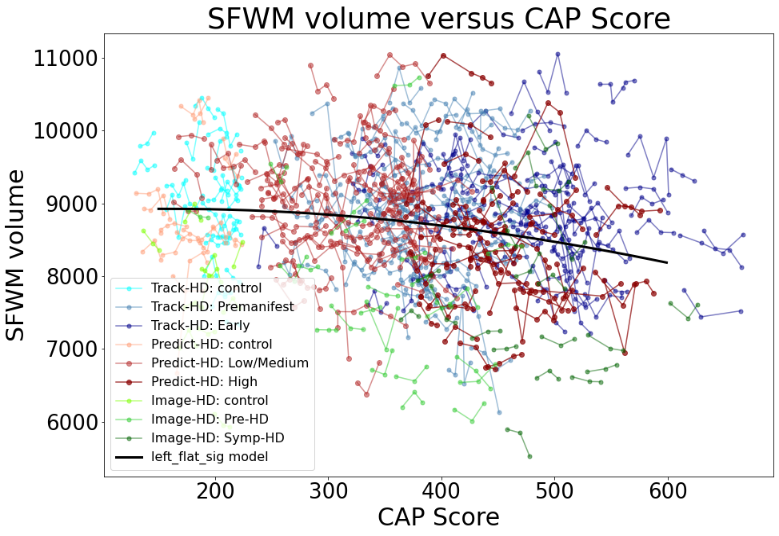

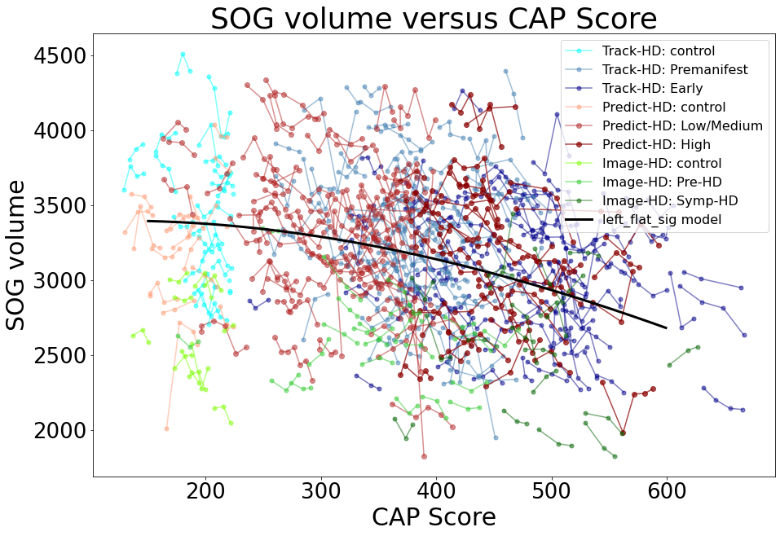

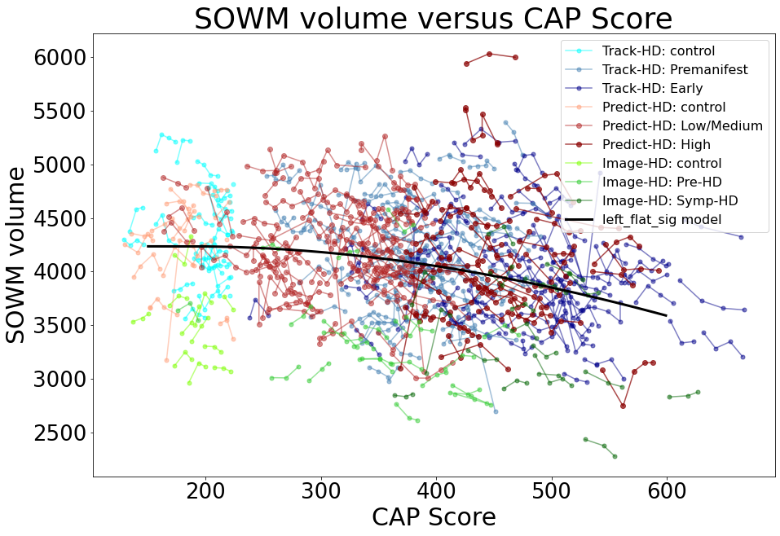

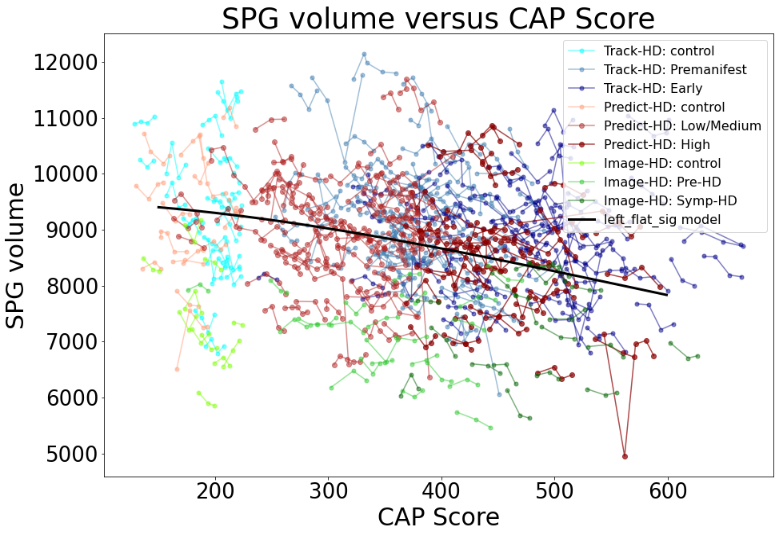

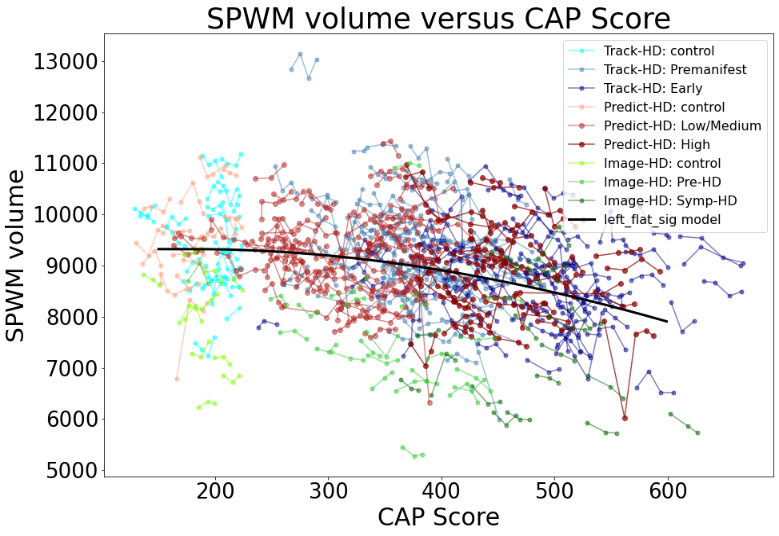

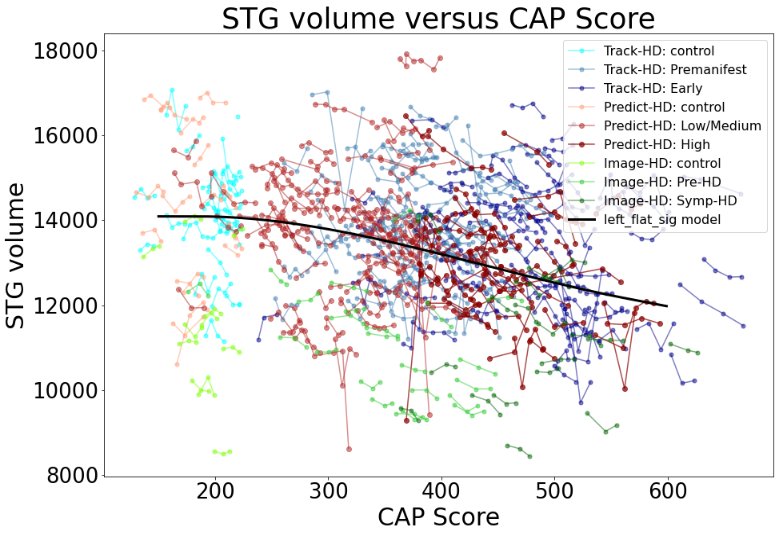

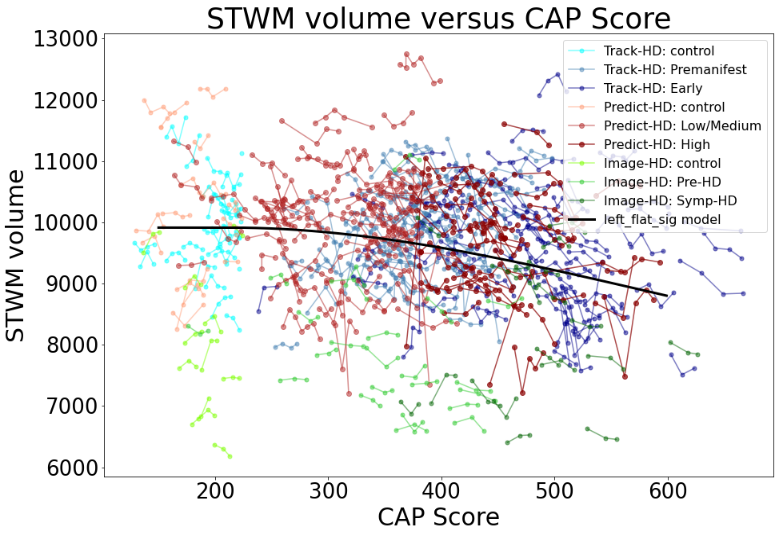


**Supplemental Figure 1: Individual longitudinal volumetric data (“spaghetti plots”) for additional brain regions**. Covariate with Intracranial Volume only (CAG expansion positive and Controls < age 40). (Datasets appear in the following order: Track-HD: control, Track-HD: Premanifest, Track-HD: Early, Predict-HD: control, Predict-HD: Low/Medium, Predict-HD: High, Image-HD: control, Image-HD: Pre-HD, Image-HD: Symp-HD, Left_flat_sig model).


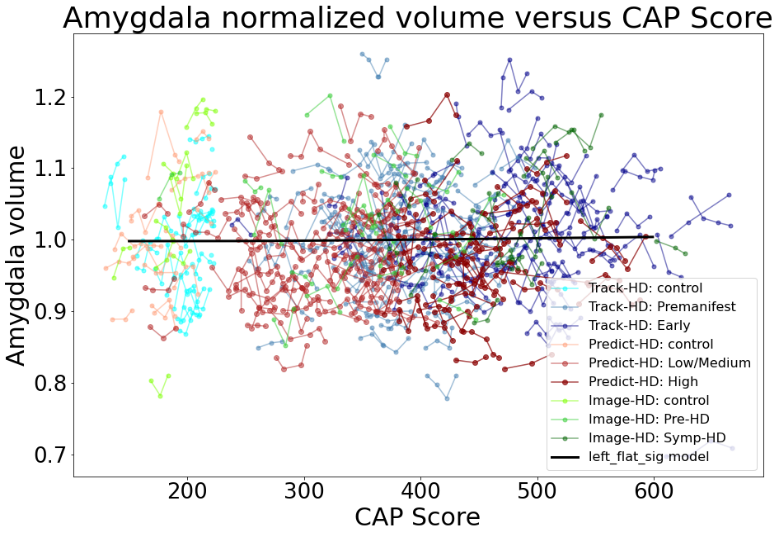

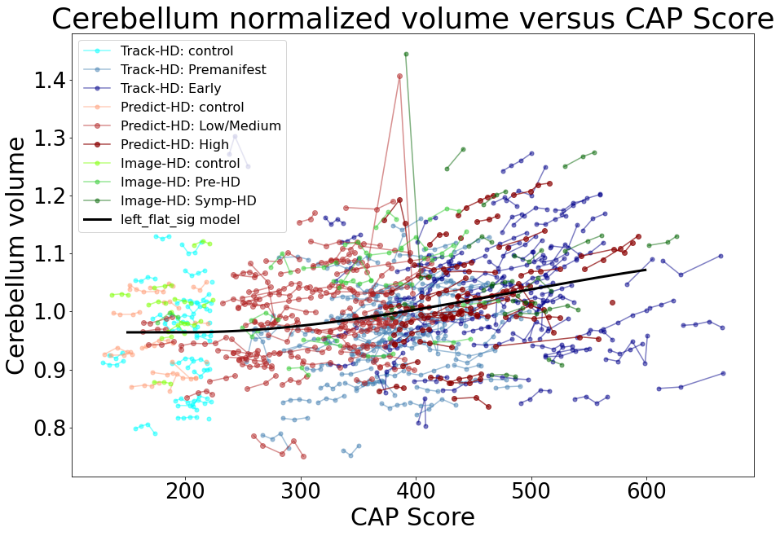

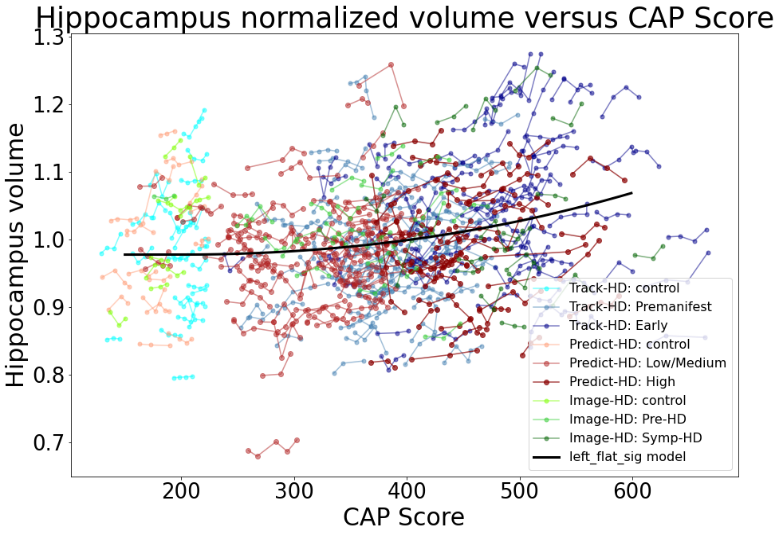

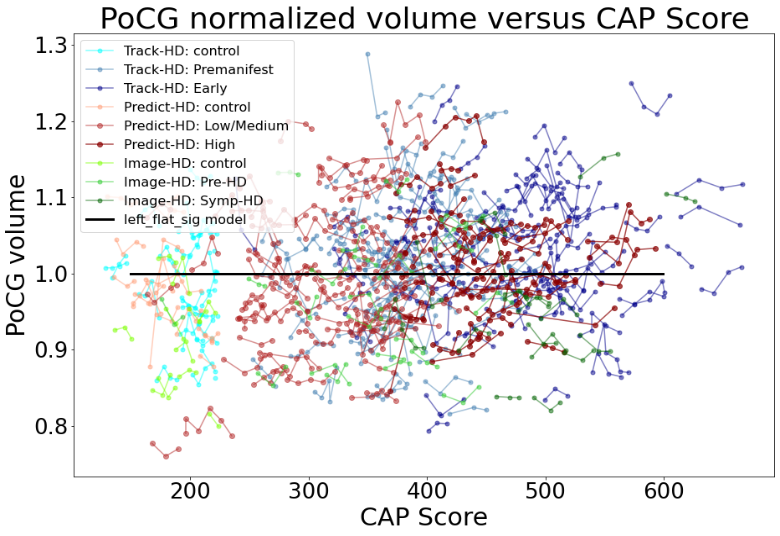

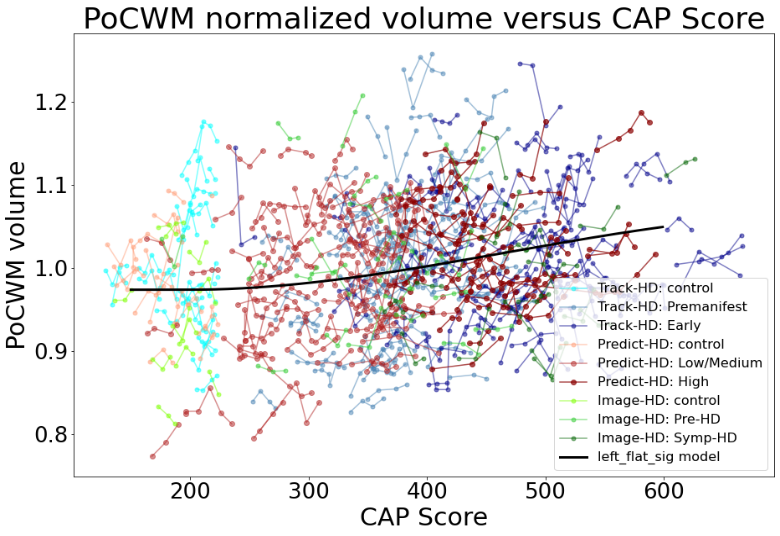

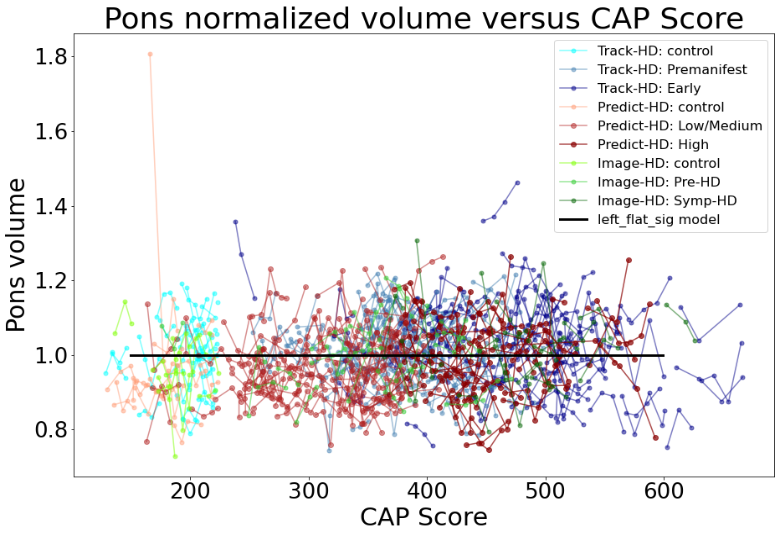

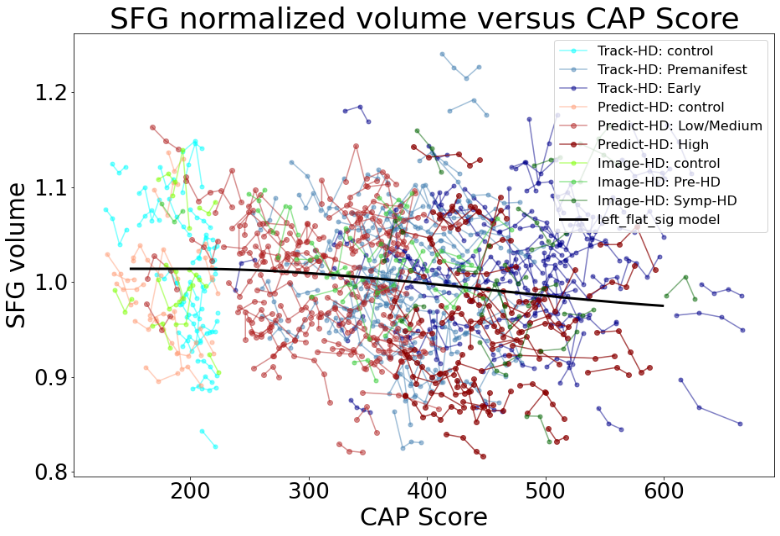

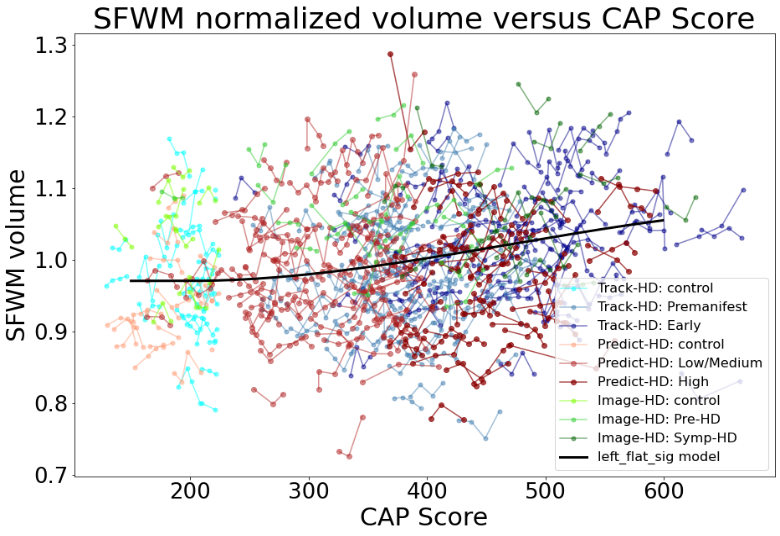

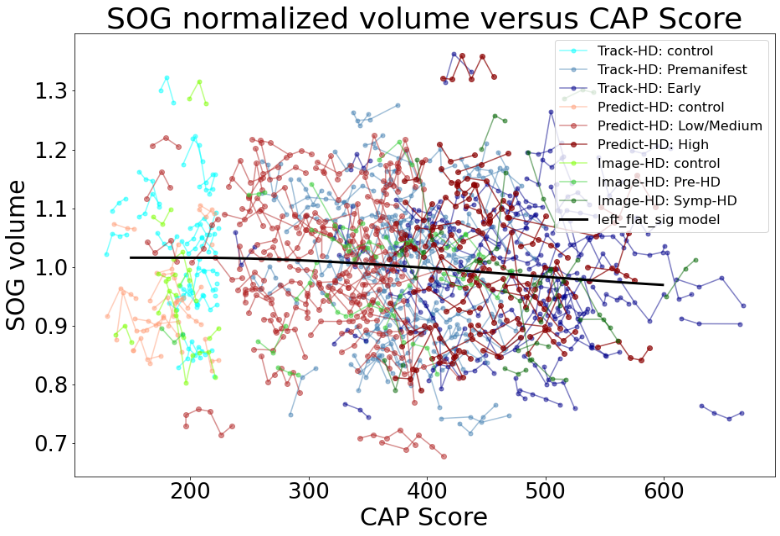

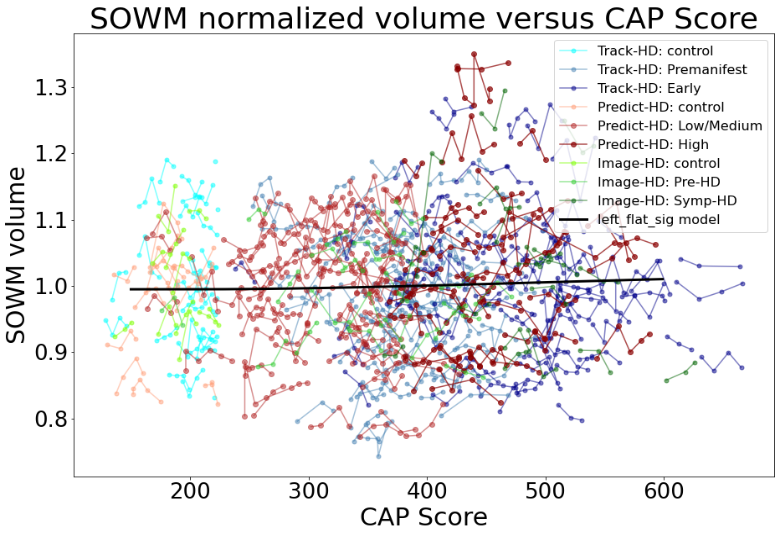

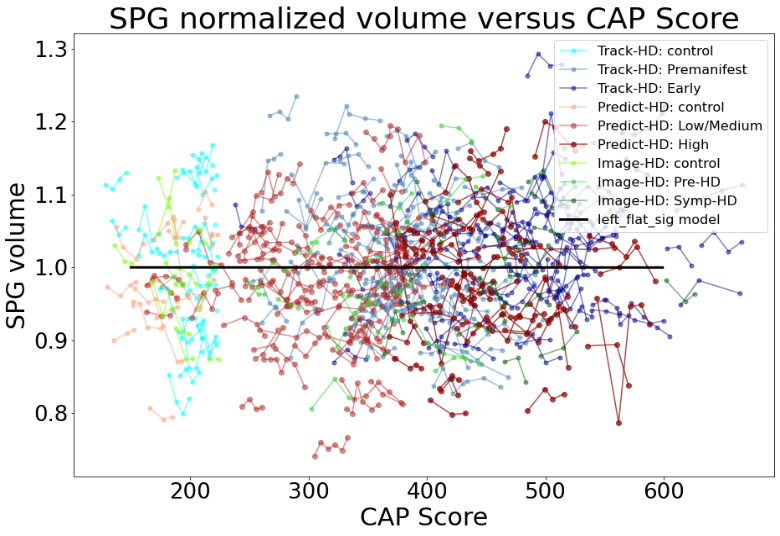

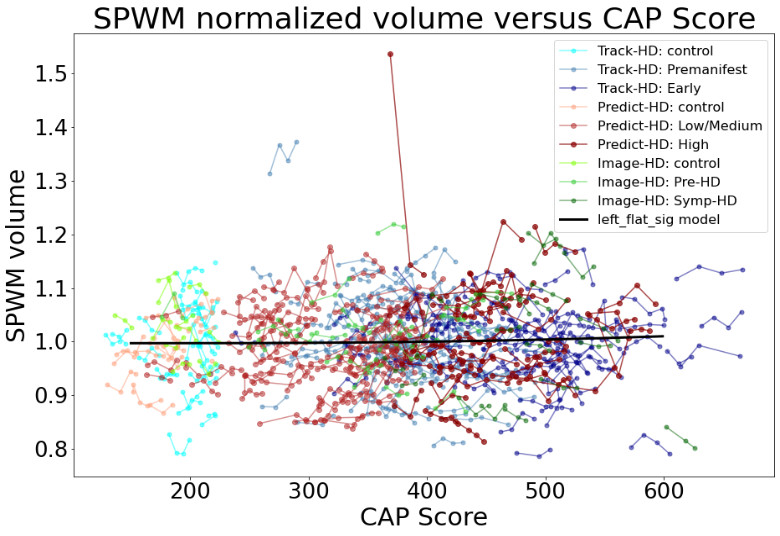

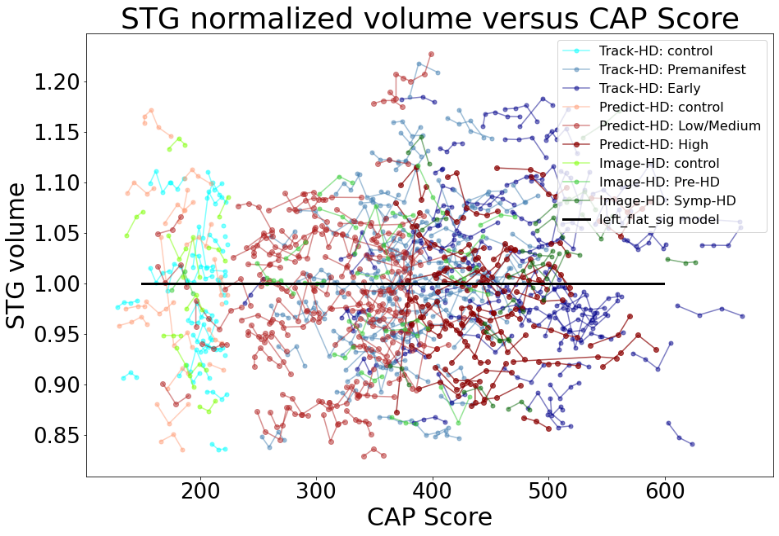

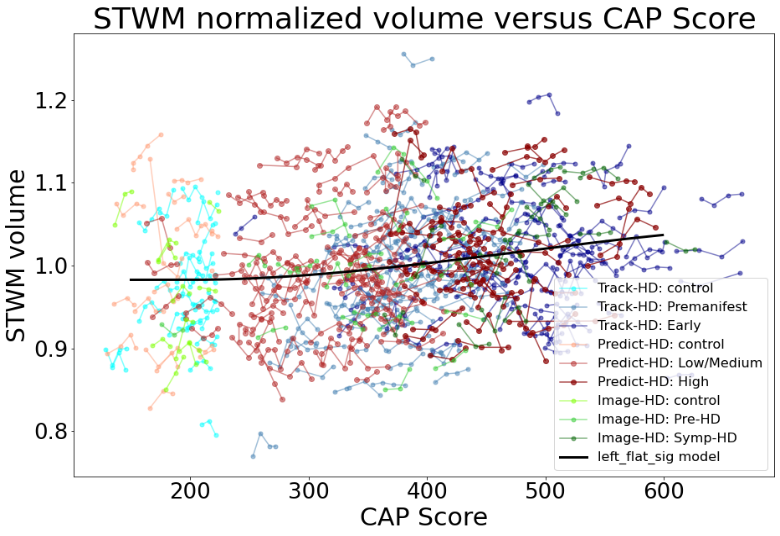


**Supplemental Figure 2: Individual longitudinal volumetric data (“spaghetti plots”) for additional regions.** Covariate with intracranial volume and normalized by whole brain volume (CAG expansion positive and Controls < age 40). (Refer to Supplemental Figure 1 for complete dataset list).


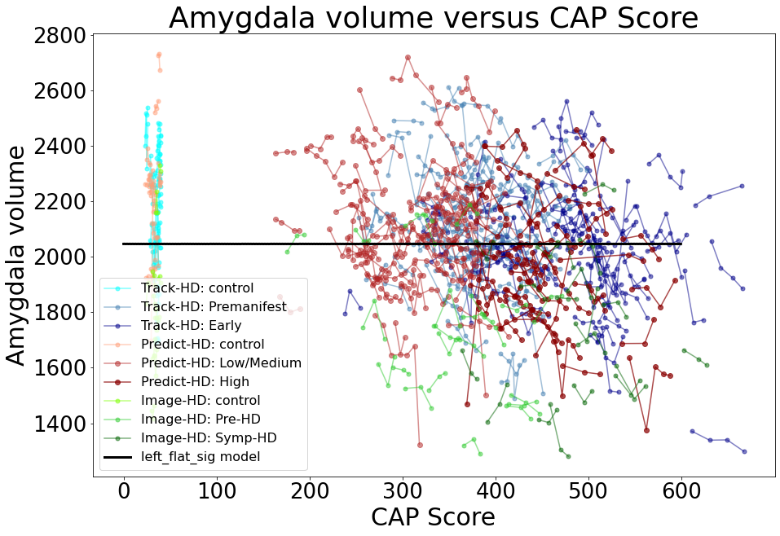

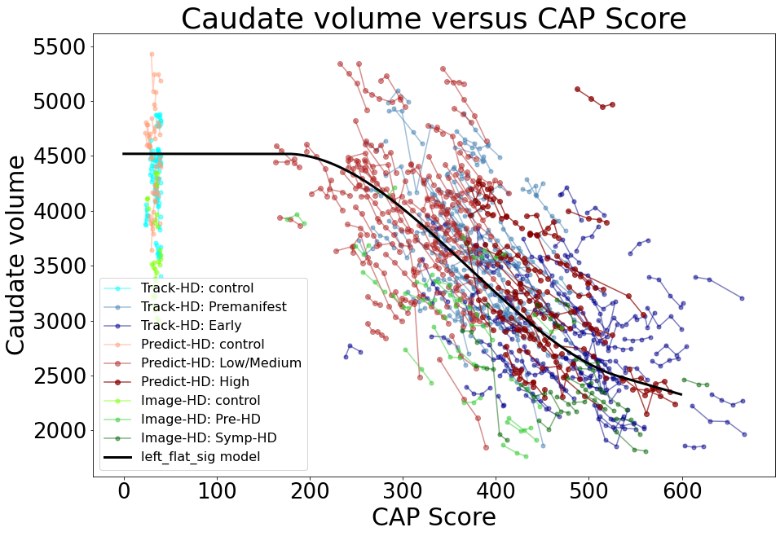

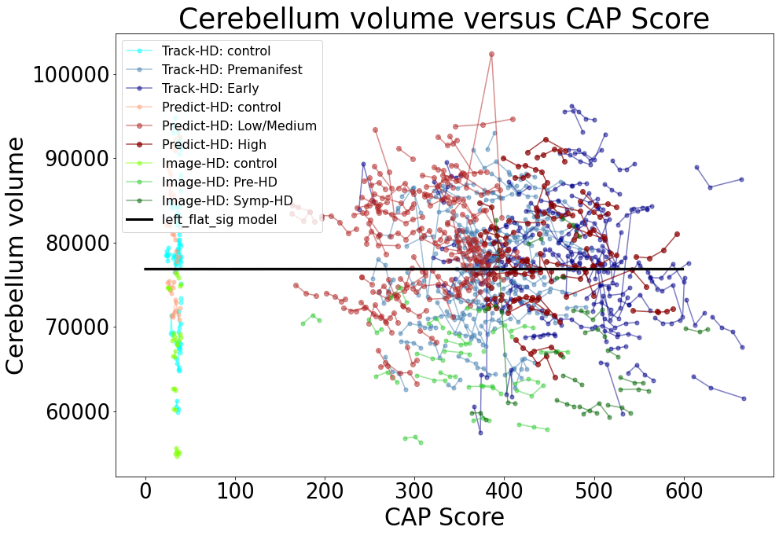

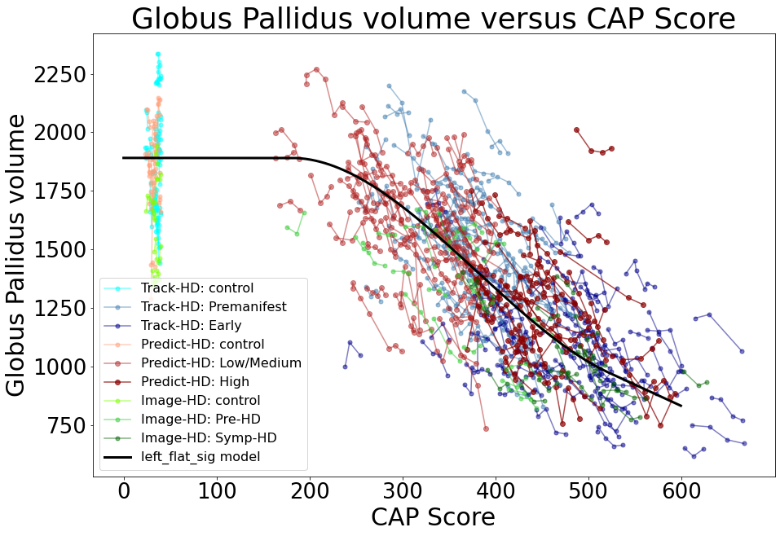

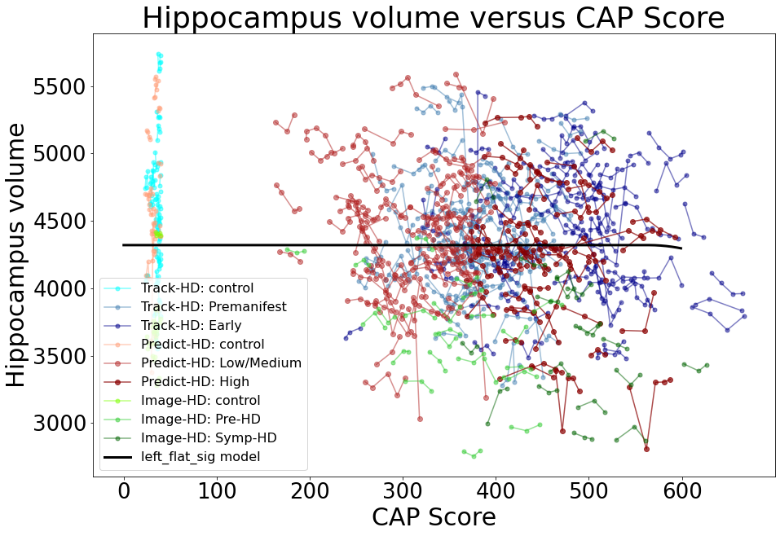

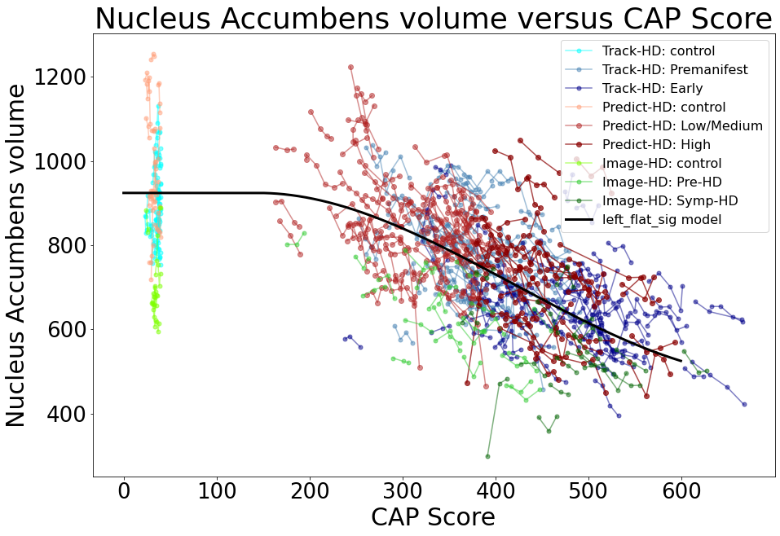

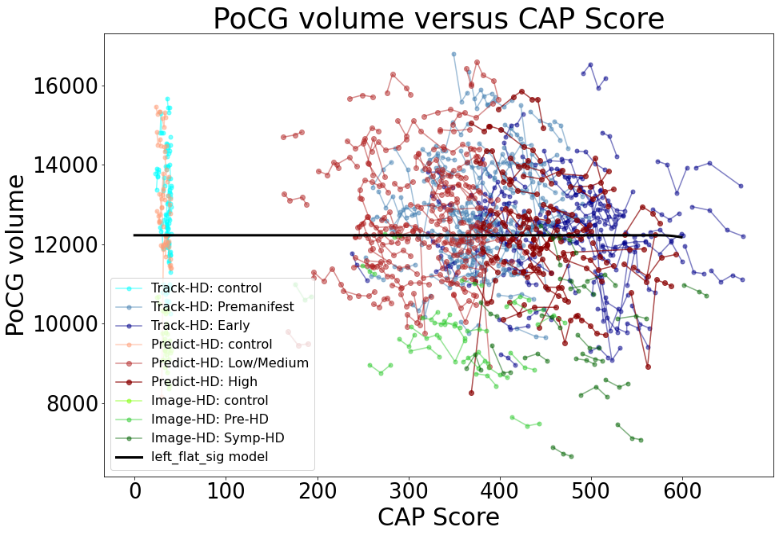

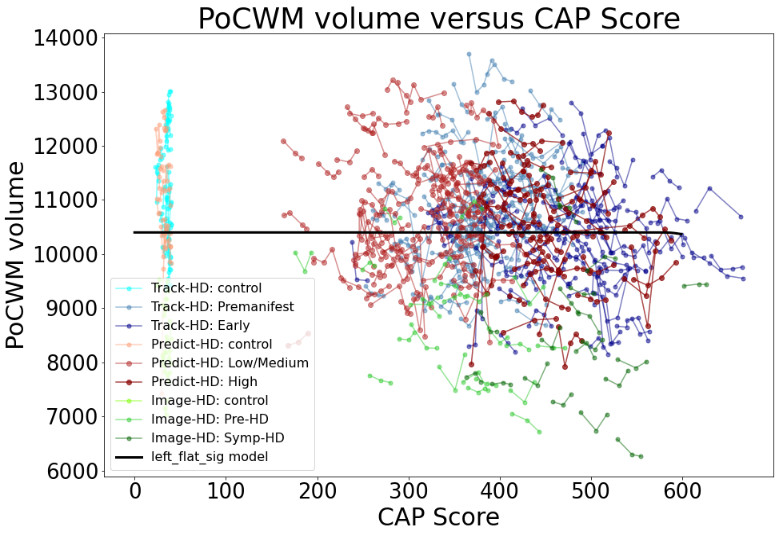

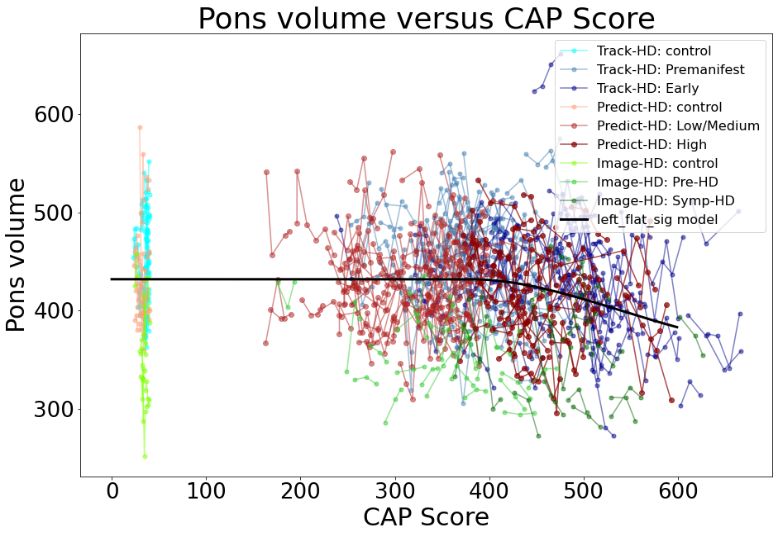

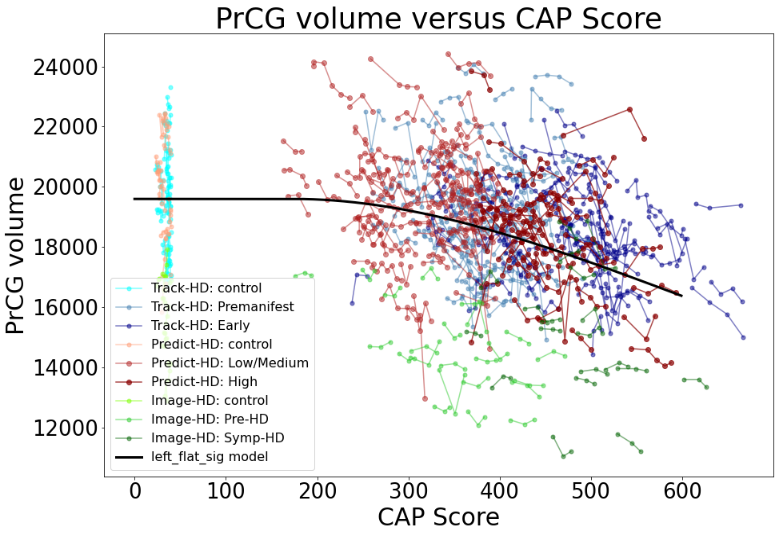

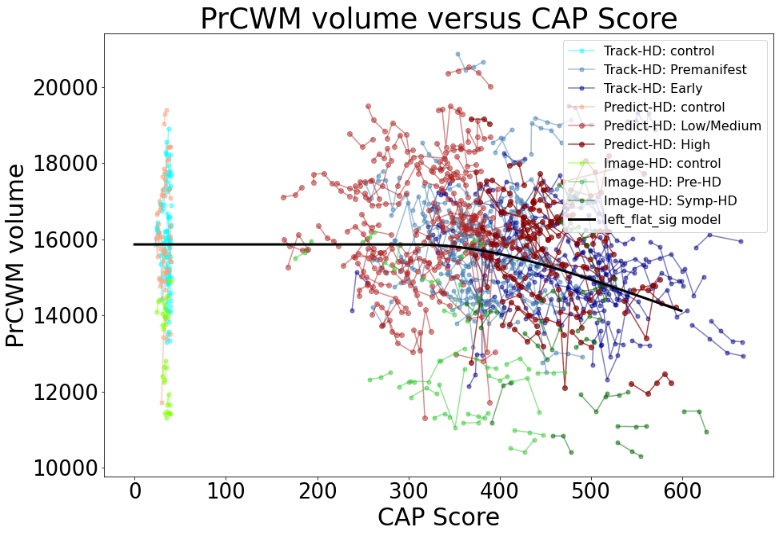

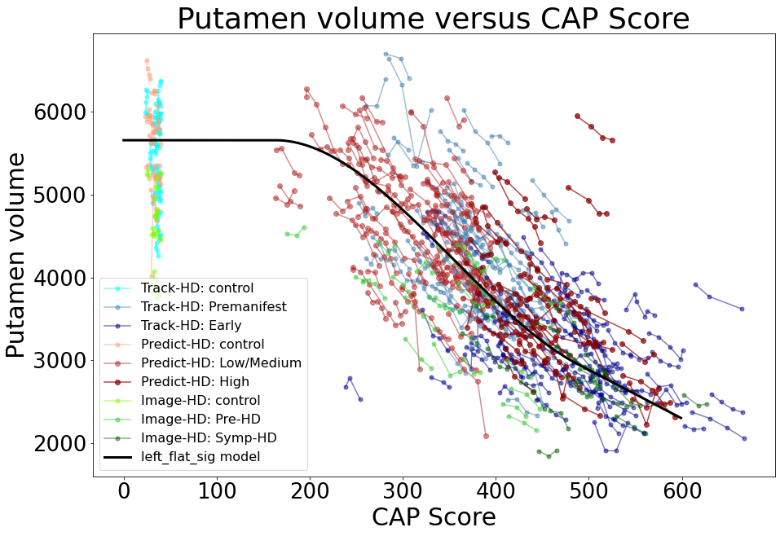

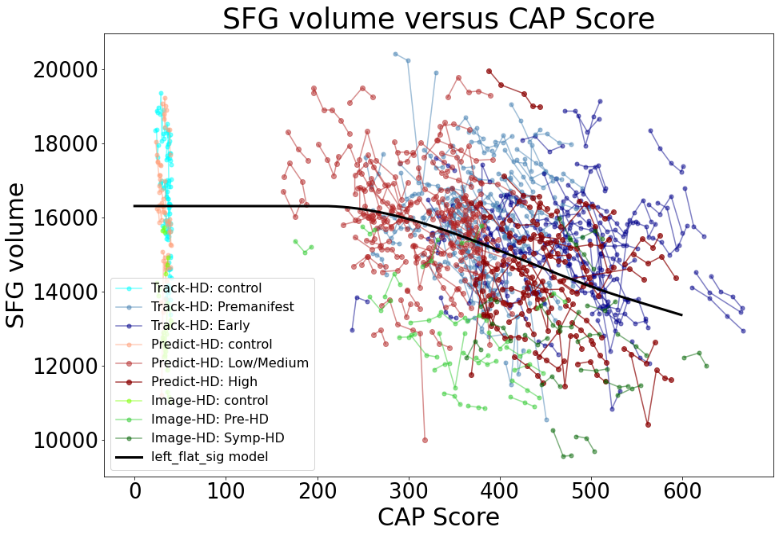

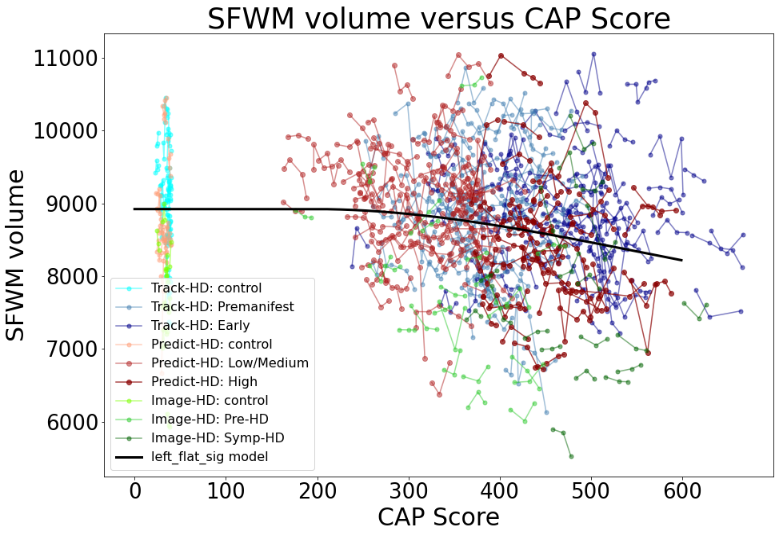

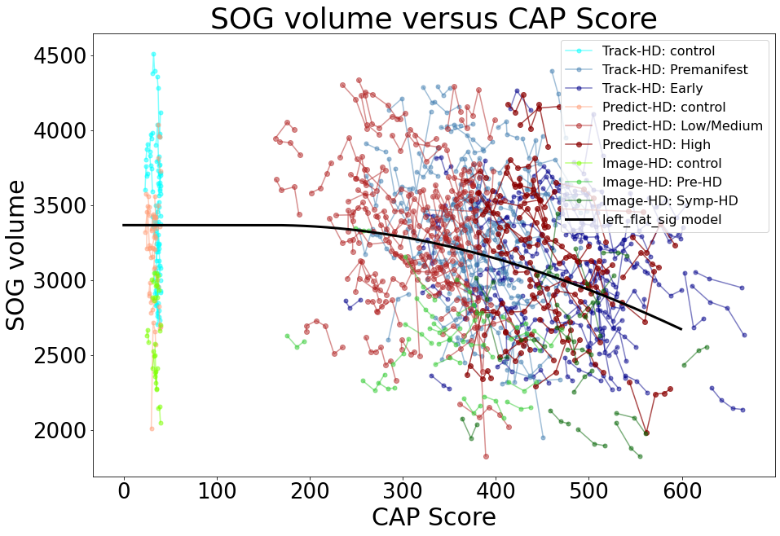

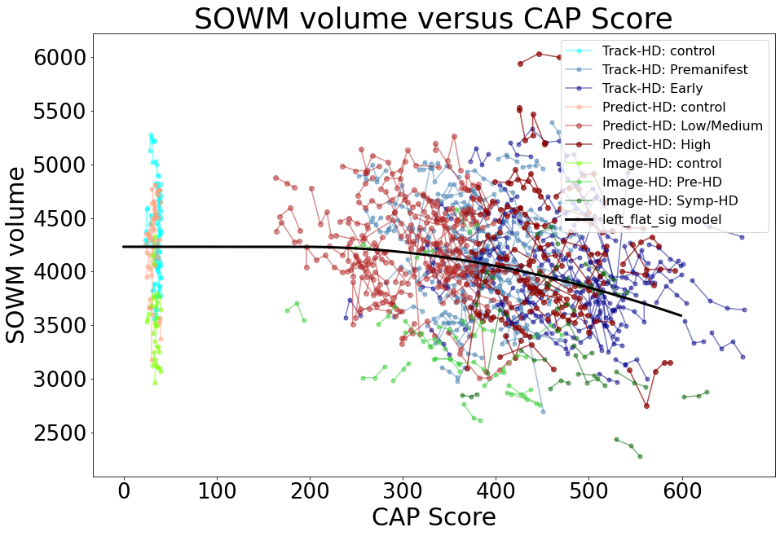

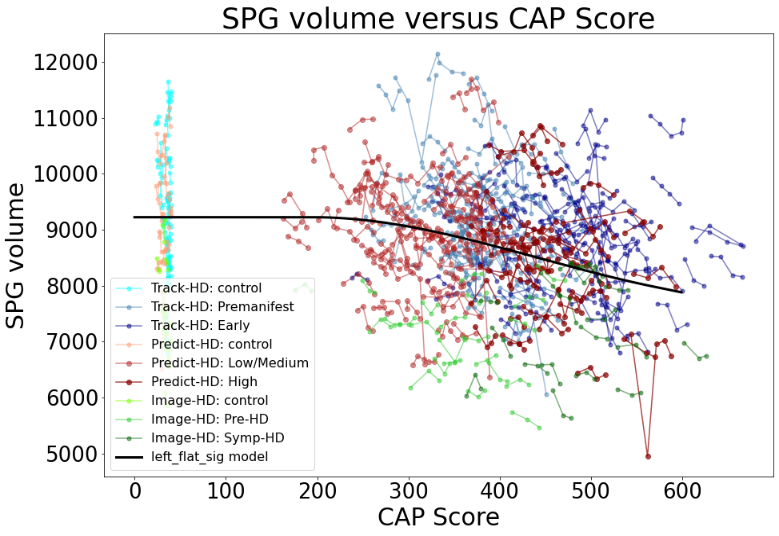

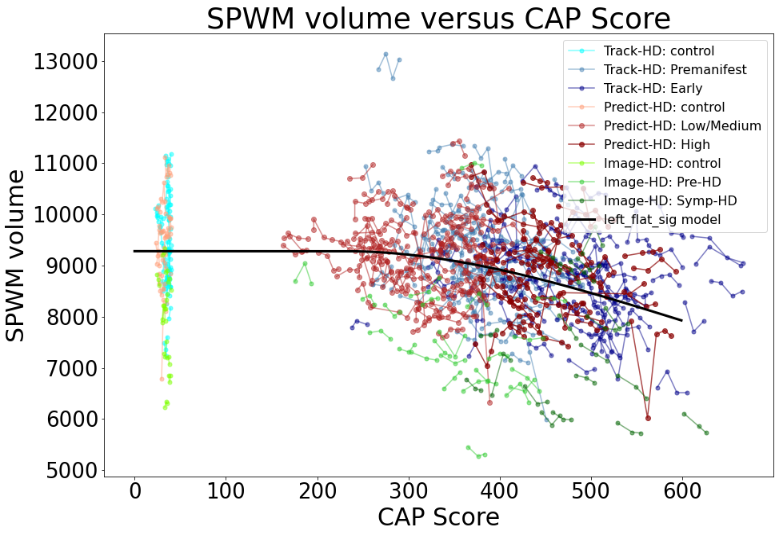

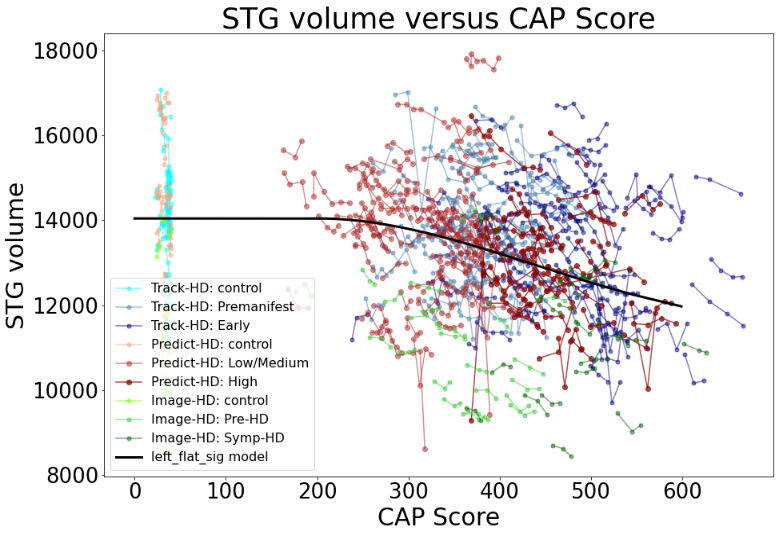

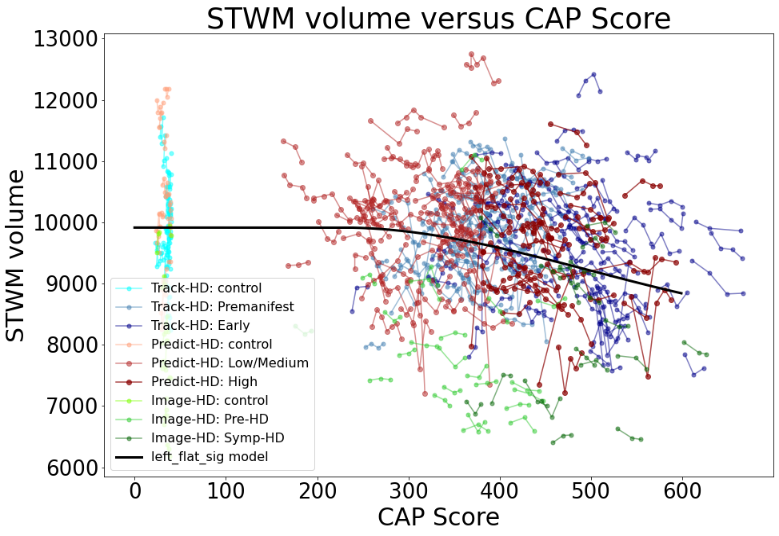

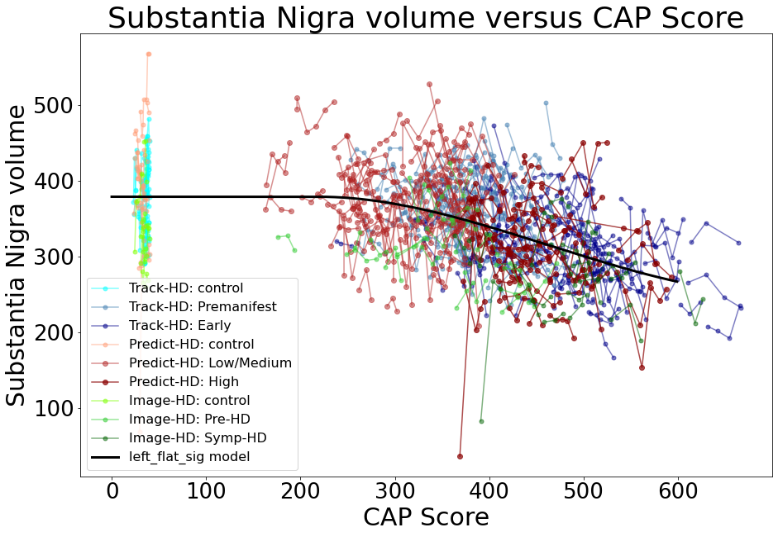

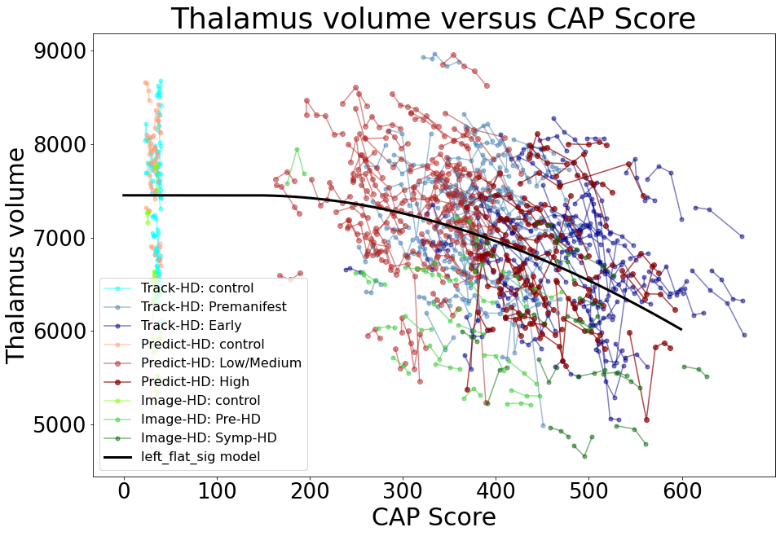


**Supplemental Figure 3: Individual longitudinal volumetric data (“spaghetti plots”) for brain regions.** Covariate intracranial volume only, analyses with control CAP = age (CAG expansion positive plus Controls age < 40). (Refer to Supplemental Figure 1 for complete dataset list).

| A. Un-normalized volumes | | | | B. Normalized Volumes | | | |
| --- | --- | --- | --- | --- | --- | --- | --- |
| Structure | **t0** | **Standard dev** | **p-value** | S**tructure** | **t0** | **Standard dev** | **p-value** |
| Caudate | 137.61 | 14.92 | 0 | **Putamen** | 139.34 | 15.62 | 0 |
| Putamen | 140.48 | 10.73 | 0 | **Caudate** | 150.38 | 22.19 | 0 |
| PrCWM | 170.99 | 59.12 | 0.016 | **NA** | 187.76 | 23.74 | 0.008 |
| GP | 181.03 | 15.37 | 0 | **PrCWM** | 210.95 | 42.58 | 0.04 |
| NA | 182.24 | 21.76 | 0 | **GP** | 216.34 | 10.20 | 0 |
| Hippo | 188.05 | 28.85 | 0.004 | **SPWM** | 282.96 | 37.57 | 0.004 |
| Thalamus | 188.81 | 39.34 | 0.028 | **Hippo** | 326.81 | 24.84 | 0.004 |
| STWM | 191.16 | 50.96 | 0.04 | **SN** | 334.69 | 38.85 | 0 |
| SN | 235.86 | 46.05 | 0 | **STG** | 534.03 | 97.81 | 0.04 |
| Amygdala | 254.58 | 44.38 | 0 |  |  |  |  |
| SFWM | 277.51 | 53.34 | 0.016 |  |  |  |  |

**Supplemental Figure 4: CAP score at slope change (t0) in increasing order for covariate intracranial volume only (A) and normalized by whole brain volume (B).** Only listed are structures for which the sigmoid model is significant over the linear one. (Abbreviations: GP: Globus Pallidus; Hippo: Hippocampus; NA: Nucleus Accumbens; PrCWM: Precentral Cortex White Matter; SN: Substantia Nigra; SFWM: Superior Frontal White Matter; SFWM: Superior Parietal White Matter; SFWM: Superior Temporal White Matter; STG: Superior Temporal Gyrus).


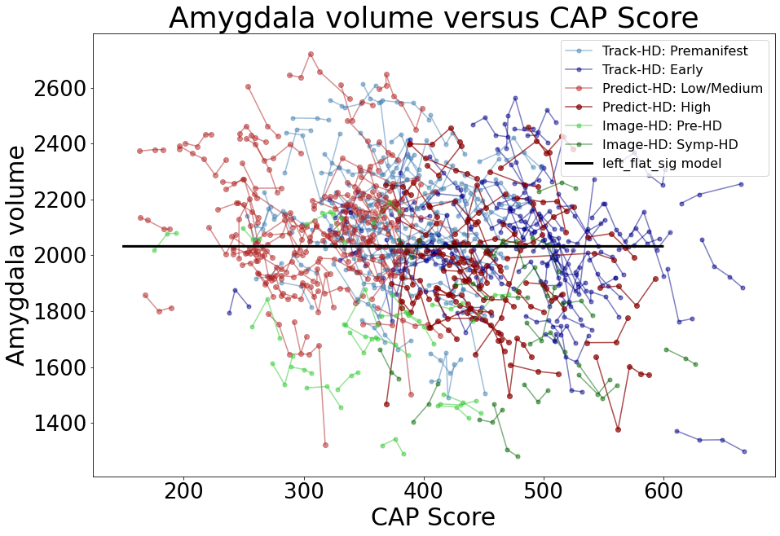

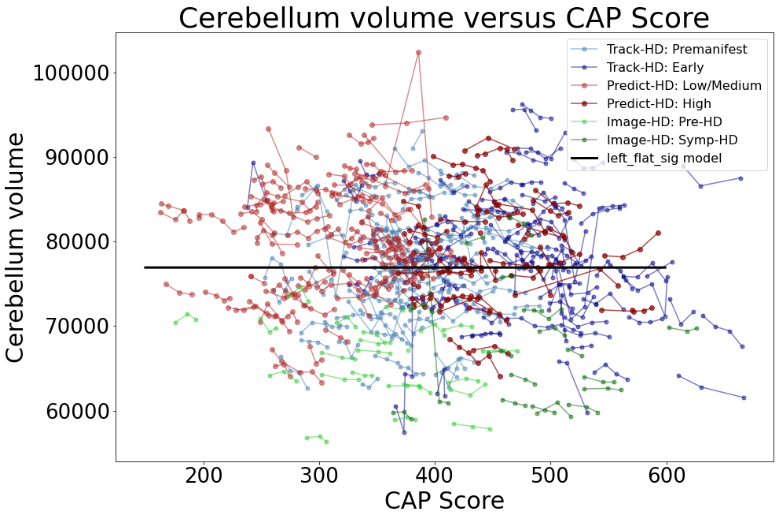

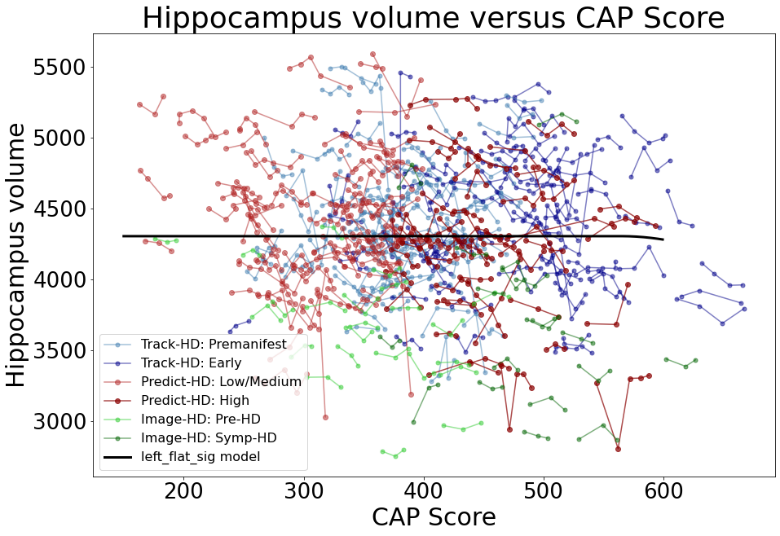

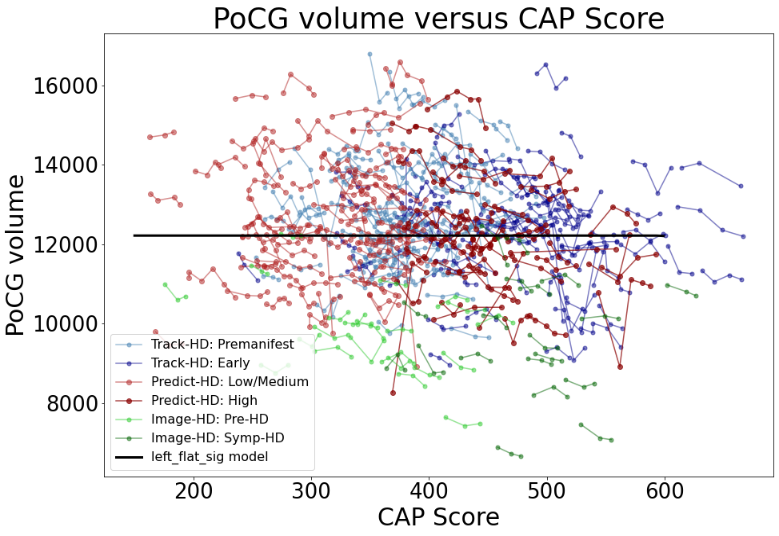

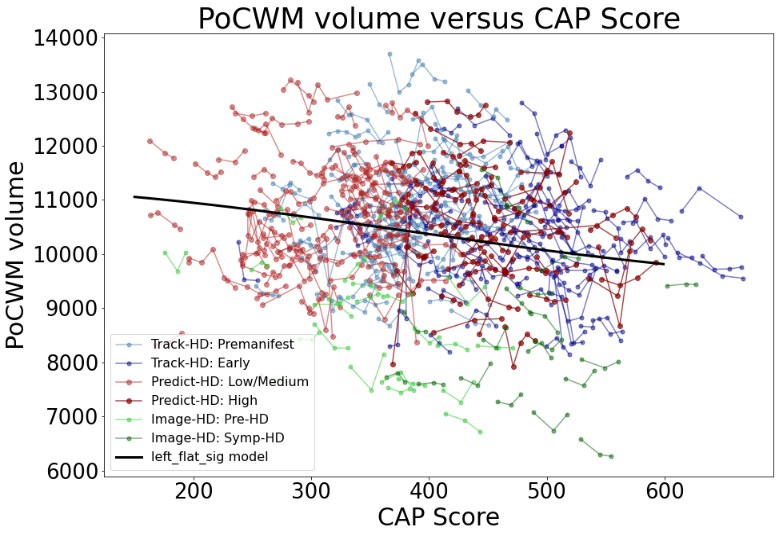

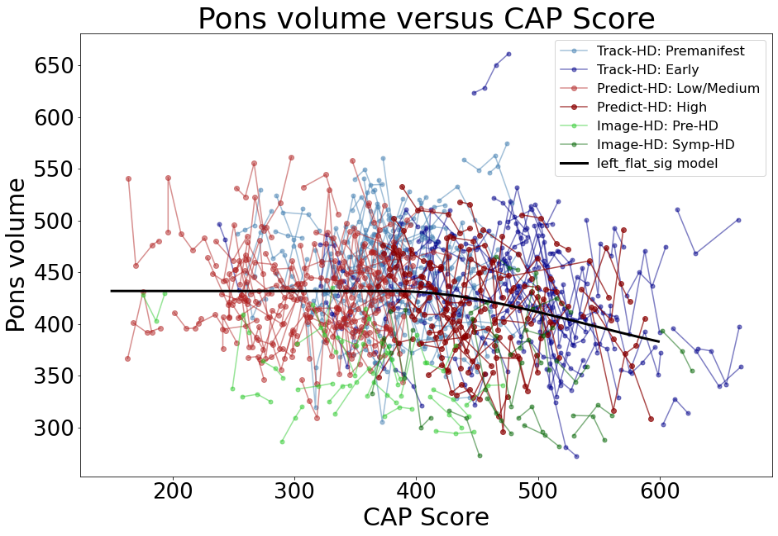

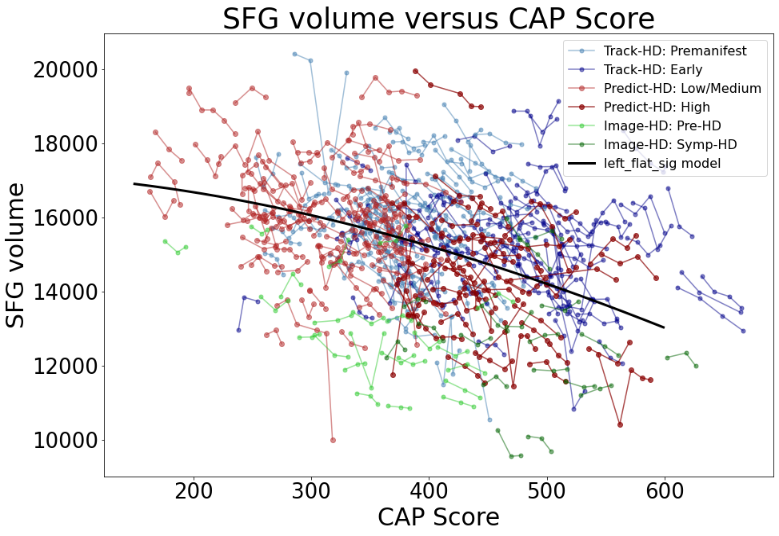

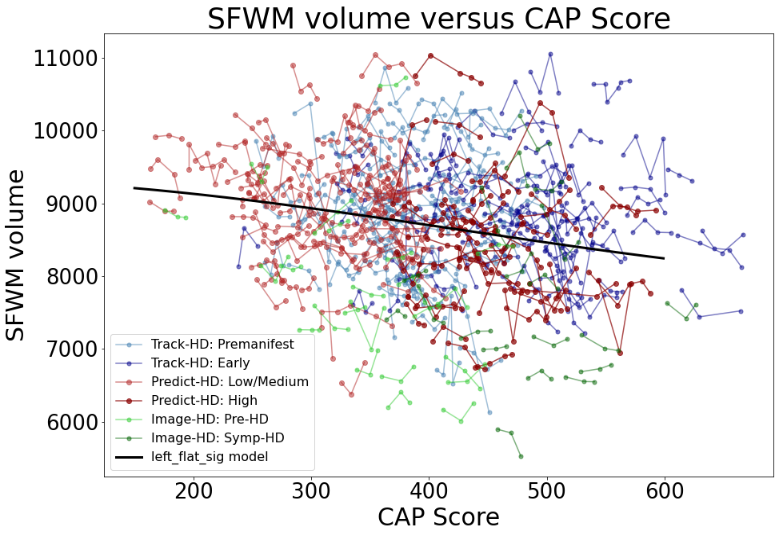

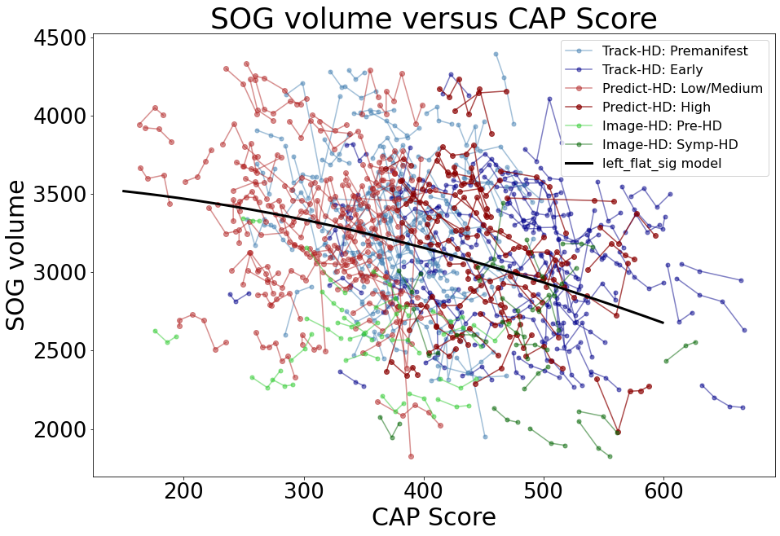

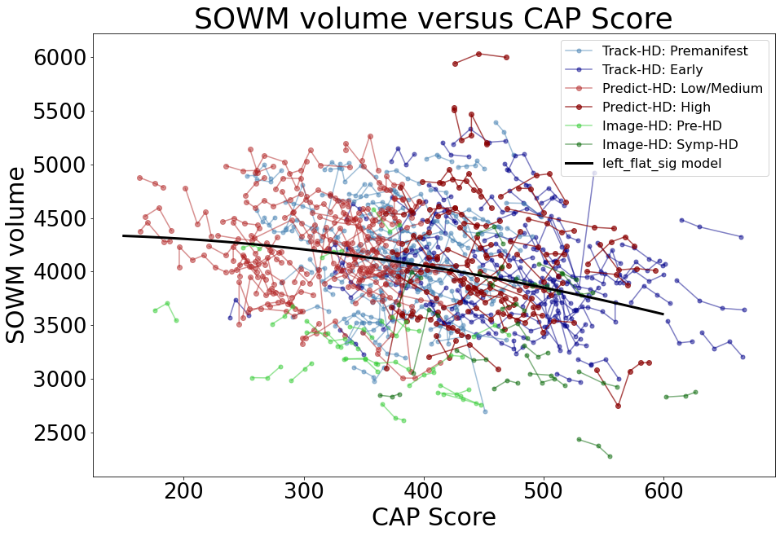

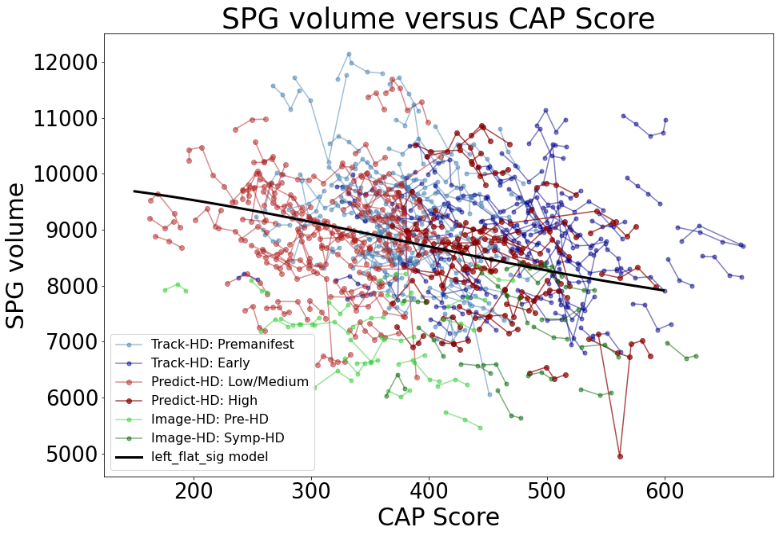

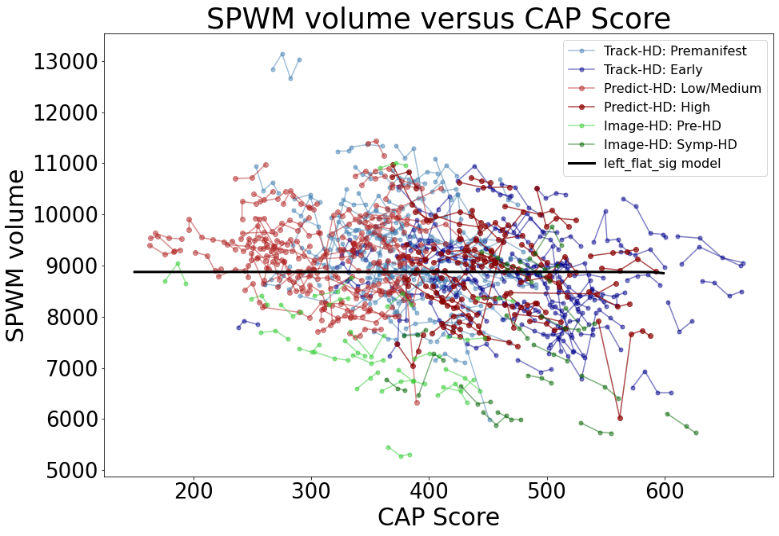

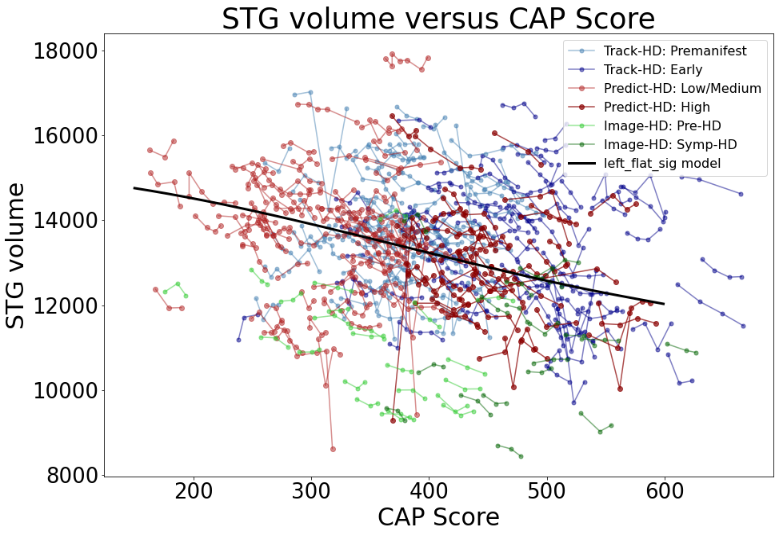

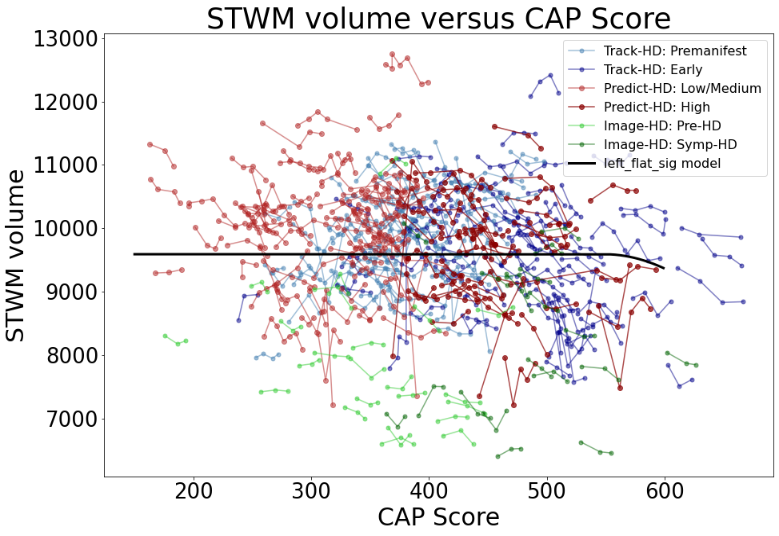


**Supplemental Figure 5: Individual longitudinal volumetric data (“spaghetti plot”) for additional regions.** Covariate intracranial volume only (CAG expansion positive only, no controls). (Refer to Figure 7 for complete dataset list).


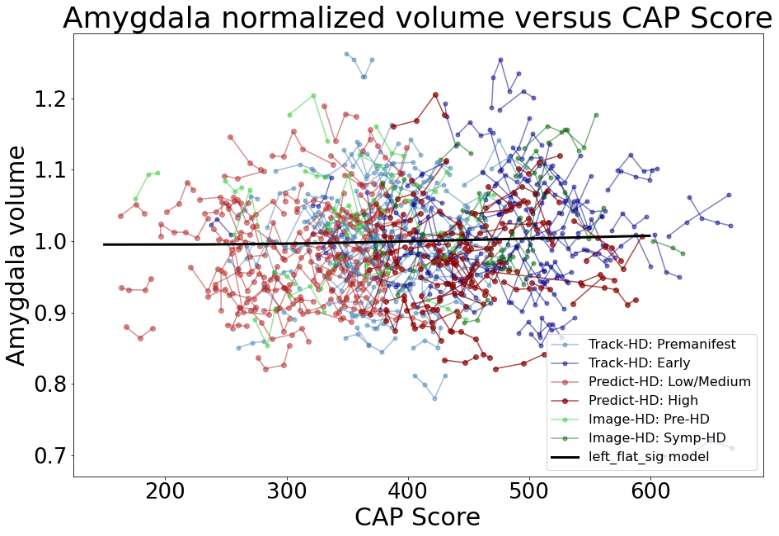

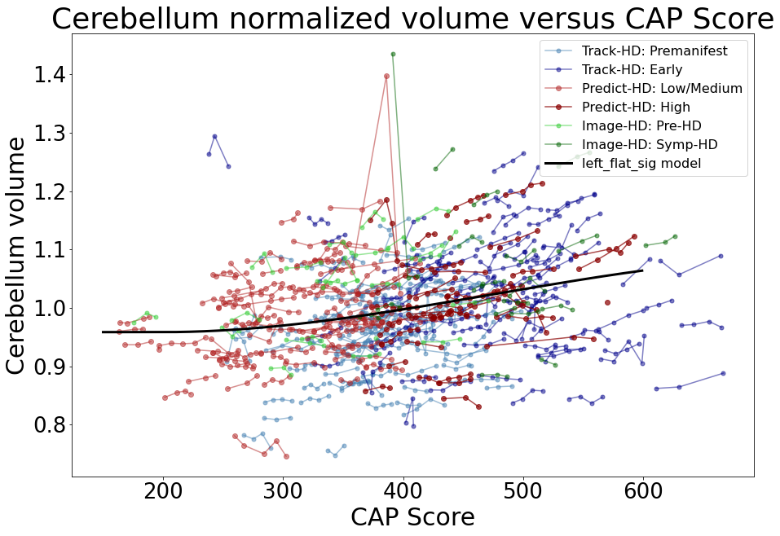

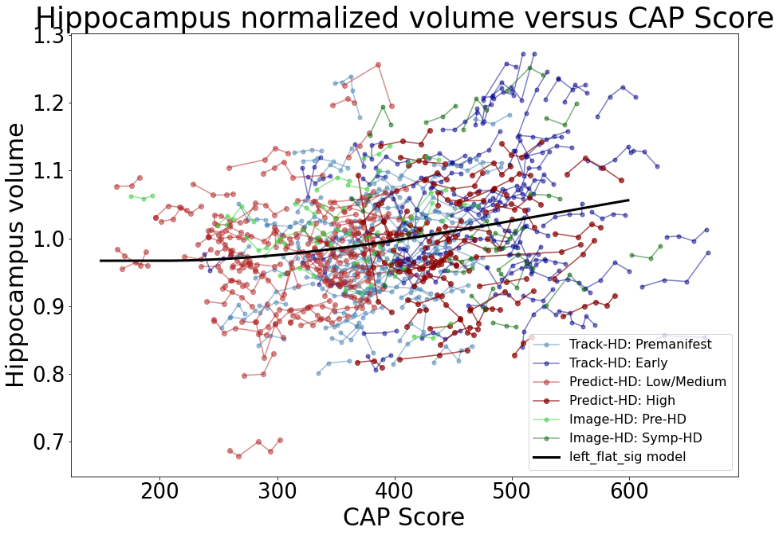

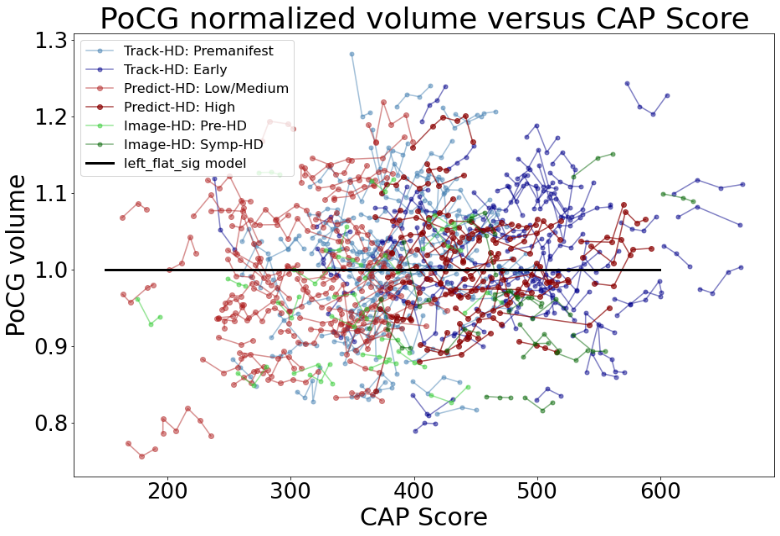

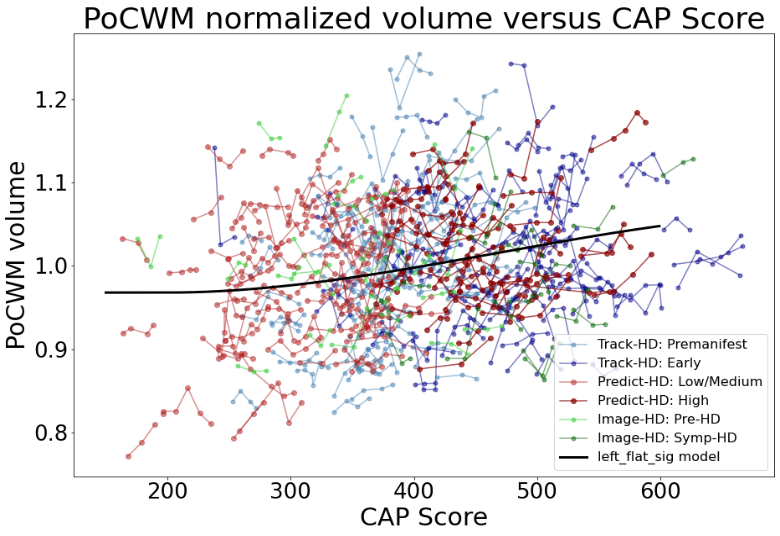

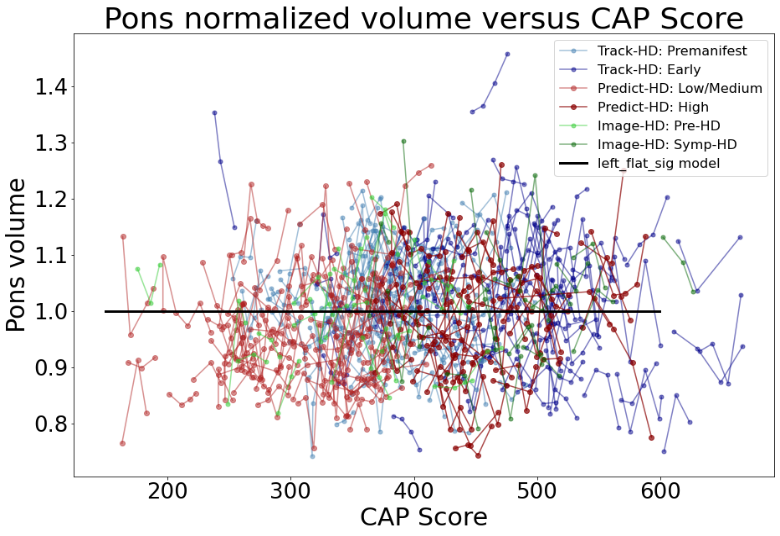

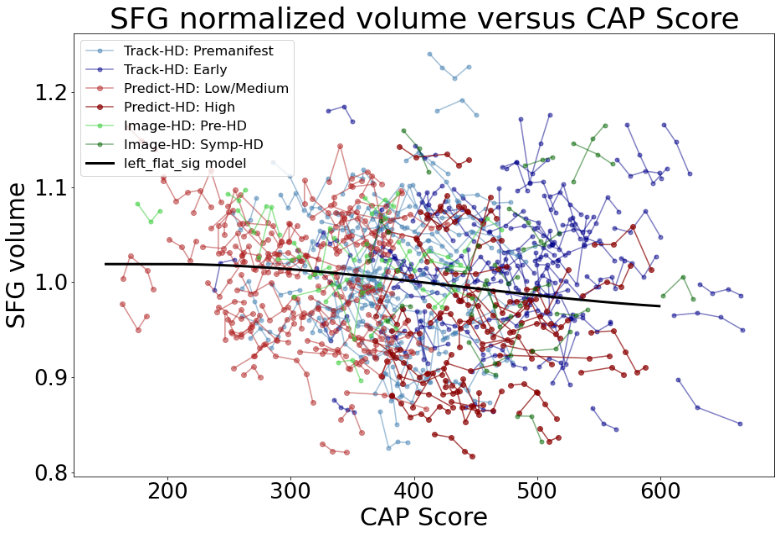

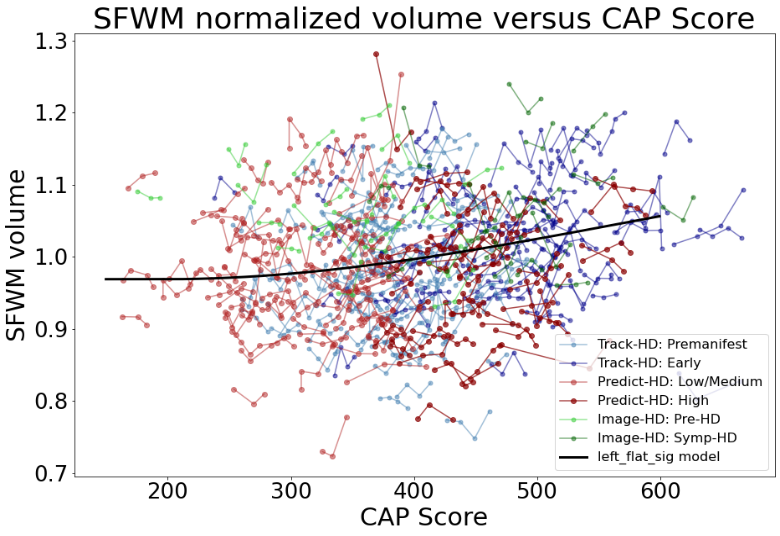

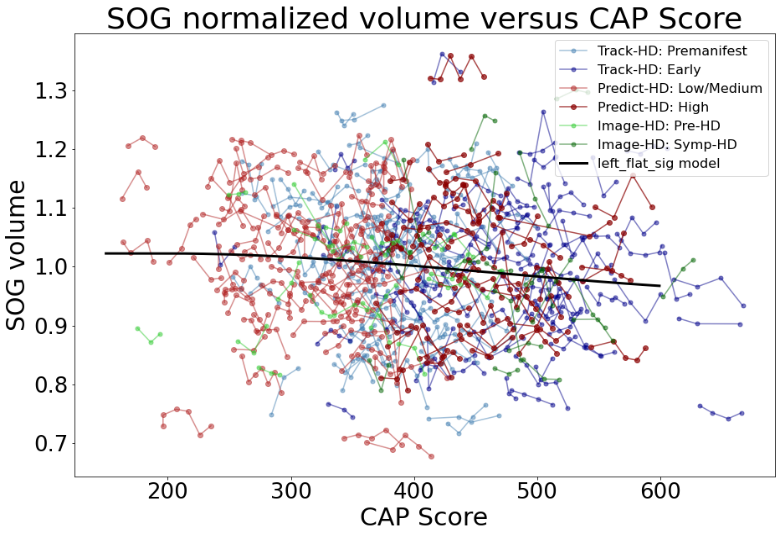

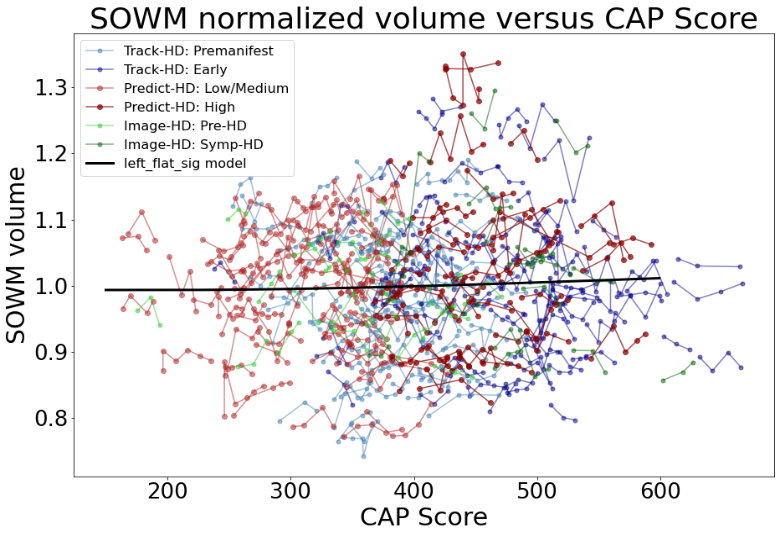

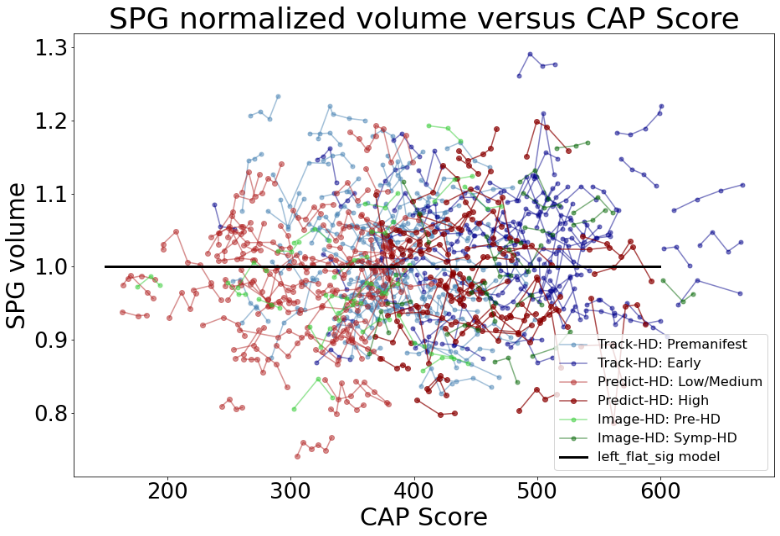

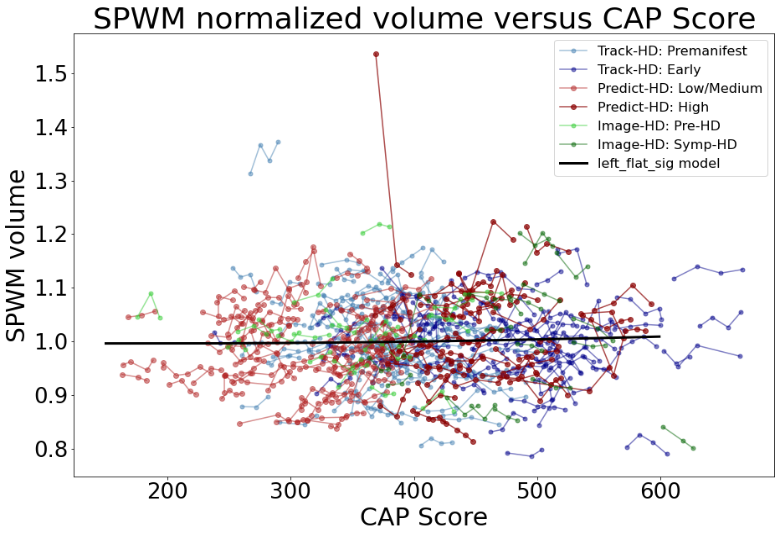

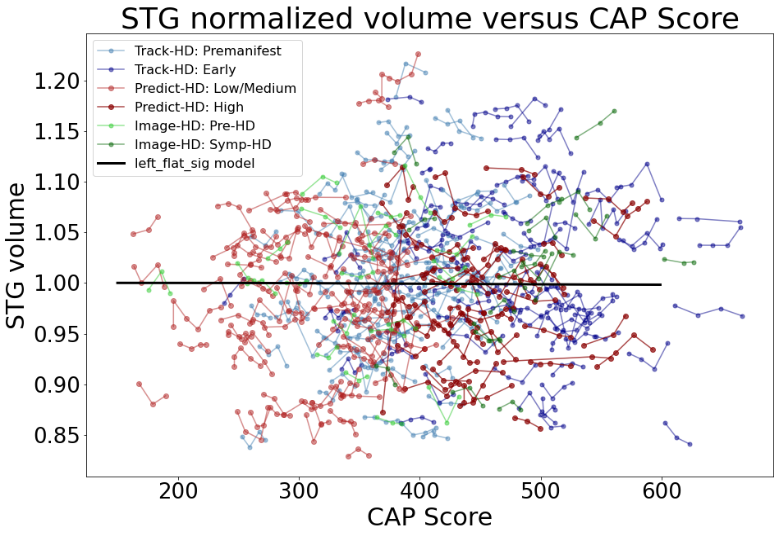

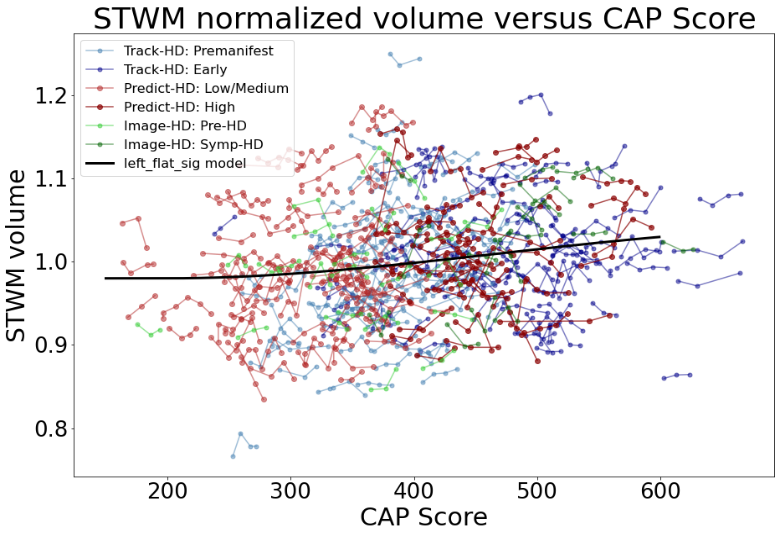


**Supplemental Figure 6: Individual longitudinal volumetric data (“spaghetti plot”) for additional regions.** Covariate intracranial volume, plus normalization by whole brain volume (CAG expansion positive only, no controls). (Refer to Figure 7 for complete dataset list).
