# Supplementary material for: Longitudinal imaging highlights preferential basal ganglia circuit atrophy in Huntington’s disease
Source: Brain Commun. 2023 Aug 18;5(5):fcad214. doi: 10.1093/braincomms/fcad214 (PMC10516592; doi:10.1093/braincomms/fcad214)
Supplement: fcad214_Supplementary_Data [file fcad214_supplementary_data.zip › Supplementary_tables.docx]

|  | **IMAGE-HD (n=42)** | | | **PREDICT-HD (n=132)** | | | **TRACK-HD (n=183)** | | |  |  |
| --- | --- | --- | --- | --- | --- | --- | --- | --- | --- | --- | --- |
| **Variable** | **Mean** | **SD** | **Range** | **Mean** | **SD** | **Range** | **Mean** | **SD** | **Range** | **Χ^2^ (df)** | **p** |
| Age | 45.6 | 10.6 | 23.9 to 65.9 | 40.3 | 12.4 | 19 to 71.6 | 45.5 | 10.1 | 18.6 to 64.1 | 17.1 (2) | <0.0001 |
| CAP* | 398.5 | 89.2 | 175.7 to 602 | 325.4 | 80.9 | 149.6 to 546.1 | 411 | 77.4 | 238.2 to 631.6 | 71.4 (2) | <0.0001 |
| CAG repeats | 42.6 | 2.0 | 39 to 47 | 42.2 | 2.7 | 38 to 55 | 43 | 2.3 | 39 to 52 | 14.1 (2) | 0.0009 |
| UHDRS- TMS | 8.5 | 10.7 | 0 to 41 | 5.4 | 5.5 | 0 to 36 | 12.3 | 13.1 | 0 to 47 | 16.6 (2) | <0.0001 |
| UHDRS-TFC |  |  |  | 12.9 | 0.5 | 10 to 13 | 12 | 1.6 | 7 to 13 | 31.2 (1) | <0.0001 |
| SDMT | 46.8 | 13.1 | 18 to 74 | 52 | 11 | 24 to 76 | 43.3 | 13.7 | 12 to 80 | 32.5 (2) | <0.0001 |
| BDI | 6.4 | 6.8 | 0 to 26 | 6.3 | 7.4 | 0 to 43 |  |  |  | 0.1 (1) | 0.770 |
| Sex, male (%) | 21 (50.0%) | | | 42 (31.8%) | | | 86 (47.0%) | | | 8.6 (2) | 0.014 |

**Supplemental Table 1. Demographic, Genetic and Clinical characteristics of participants (n=357) at enrollment by study site.** Comparison of HD participants by study-site in the IMAGE-HD, PREDICT-HD, and TRACK-HD studies. Only data from those participants whose imaging data passed the quality-control check are included. SD, Standard deviation; Χ^2^, Chi-square; df, Degrees of freedom; CAP, CAG-Age Product; UHDRS, Unified Huntington's Disease Rating Scale; TMS, Total Motor Score; TFC, Total Functional Capacity; SDMT, Symbol-Digit Modalities Test; BDI, Beck Depression Inventory. TFC data were not reported by IMAGE-HD and BDI data were not reported by TRACK-HD. Χ^2^ values and corresponding p-values are from the Kruskal-Wallis test for continuous variables and the Χ^2^ test for the nominal variable (sex). *Arbitrary value for CAP of controls calculated as described in the text

**Supplemental Table 2**

| Dataset | Pre Segmentation Quality Control | | Post Segmentation Quality Control | |
| --- | --- | --- | --- | --- |
|  | # Subjects | # Scans | # Subjects | # Scans |
| Predict-HD | 670 | 1484 | 655 | 1444 |
| Track-HD | 392 | 1397 | 386 | 1263 |
| Image-HD | 112 | 283 | 107 | 259 |

**Suppl Table 2A:** Number of subjects and scans from the Predict-HD, Track-HD and Image-HD datasets before and after Segmentation Quality Control Elimination (All subjects).

| Dataset | Pre Segmentation Quality Control | | Post Segmentation Quality Control | |
| --- | --- | --- | --- | --- |
|  | # Subjects | # Scans | # Subjects | # Scans |
| Predict-HD | 203 | 829 | 194 | 792 |
| Track-HD | 327 | 1292 | 283 | 1099 |
| Image-HD | 77 | 231 | 63 | 189 |

**Suppl Table 2B:** Number of subjects and scans from the Predict-HD, Track-HD and Image-HD datasets before and after Segmentation Quality Control Elimination (Only subjects with at least 3 scans).
